# Self-supervised deep learning encodes high-resolution features of protein subcellular localization

**DOI:** 10.1038/s41592-022-01541-z

**Published:** 2022-07-25

**Authors:** Hirofumi Kobayashi, Keith C. Cheveralls, Manuel D. Leonetti, Loic A. Royer

**Affiliations:** Chan Zuckerberg Biohub, San Francisco, CA USA

**Keywords:** Machine learning, Data mining

## Abstract

Explaining the diversity and complexity of protein localization is essential to fully understand cellular architecture. Here we present cytoself, a deep-learning approach for fully self-supervised protein localization profiling and clustering. Cytoself leverages a self-supervised training scheme that does not require preexisting knowledge, categories or annotations. Training cytoself on images of 1,311 endogenously labeled proteins from the OpenCell database reveals a highly resolved protein localization atlas that recapitulates major scales of cellular organization, from coarse classes, such as nuclear and cytoplasmic, to the subtle localization signatures of individual protein complexes. We quantitatively validate cytoself’s ability to cluster proteins into organelles and protein complexes, showing that cytoself outperforms previous self-supervised approaches. Moreover, to better understand the inner workings of our model, we dissect the emergent features from which our clustering is derived, interpret them in the context of the fluorescence images, and analyze the performance contributions of each component of our approach.

## Abstract (web summary)

Cytoself is a self-supervised deep learning-based approach for profiling and clustering protein localization from fluorescence images. Cytoself outperforms established approaches and can accurately predict protein subcellular localization.

## Main

Systematic and large-scale microscopy-based cell assays are becoming an increasingly important tool for biological discovery^1,2^, playing a key role in drug screening^3,4^, drug profiling^5,6^ and for mapping the subcellular localization of the proteome^7,8^. In particular, large-scale datasets based on immuno-fluorescence or endogenous fluorescent tagging comprehensively capture localization patterns across the human^9,10^ and yeast proteome^11^. Together with recent advances in computer vision and deep learning^12^, such datasets are poised to help systematically map the cell’s spatial architecture. This situation is reminiscent of the early days of genomics, when the advent of high-throughput and -fidelity sequencing technologies was accompanied by the development of new algorithms to analyze, compare and categorize these sequences, and the genes therein. However, images pose unique obstacles to analysis. While sequences can be compared against a frame of reference (that is, genomes), there are no such references for microscopy images. Indeed, cells exhibit a wide variety of shapes and appearances that reflect a plurality of states. This rich diversity is much harder to model and analyze than, for example, sequence variability. Moreover, much of this diversity is stochastic, posing the additional challenge of separating information of biological relevance from irrelevant variance. The fundamental computational challenge posed by image-based screens is therefore to extract well-referenced vectorial representations that faithfully capture only the relevant biological information and allow for quantitative comparison, categorization and biological interpretation of protein localization patterns.

Previous approaches to classify and compare images have relied on engineered features that quantify different aspects of image content, such as cell size, shape and texture^13–16^. While these features are, by design, relevant and interpretable, the underlying assumption is that all the relevant features needed to analyze an image can be identified and appropriately quantified. This assumption has been challenged by deep learning’s recent successes^17^. On a wide range of computer vision tasks such as image classification, hand-designed features cannot compete against learned features that are automatically discovered from the data themselves^18,19^. Assuming features are available, the typical approach consists of boot-strapping the annotation process by either (1) unsupervised clustering techniques^20,21^, or (2) manual curation and supervised learning^22,23^. In the case of supervised approaches, human annotators examine images and assign annotations, and once sufficient data are garnered, a machine learning model is trained in a supervised manner and later applied to unannotated data^17,18,23,24^. Another approach consists of reusing models trained on natural images to learn generic features on which supervised training can be bootstrapped^5,25,26^. While successful, these approaches suffer from potential biases, as manual annotation imposes our own preconceptions. Overall, the ideal algorithm should not rely on human knowledge or judgments, but instead automatically synthesize features and analyze images without a priori assumptions, that is, solely on the basis of the images themselves.

Recent advances in computer vision and machine learning have shown that forgoing manual labeling is possible and nears the performance of supervised approaches^27,28^. Instead of annotating datasets, which is inherently nonscalable and labor-intensive, self-supervised models can be trained from large uncurated datasets^11,29–32^. Self-supervised models are trained by formulating an auxiliary pretext task, typically one that withholds parts of the data and instructs the model to predict them^33^. This works because the task-relevant information within a piece of data is often distributed over multiple observed dimensions^30^. For example, given the picture of a car, we can recognize the presence of a vehicle even if many pixels are hidden, perhaps even when half of the image is occluded. Now, consider a large dataset of pictures of real-world objects (for example, ImageNet^34^). Training a model to predict hidden parts from these images forces it to identify their important features^32^. Once trained, the vectorial representations that emerge from pretext tasks capture the important features of the images, and can be used for comparison and categorization.

Here, we present the development, validation and use of cytoself, a deep learning-based approach for fully self-supervised protein localization profiling and clustering. The key innovation is a pretext task that ensures that the localization features that emerge from different images of the same protein are helpful to distinguish the microscopy images of that protein from the images of other proteins in the dataset. We demonstrate the ability of cytoself to reduce images to feature profiles characteristic of protein localization, validate their use to predict protein assignment to organelles and protein complexes, and compare the performance of cytoself with previous image featurization approaches.

## Results

### A robust and comprehensive image dataset

A prerequisite to our deep-learning approach is a collection of high-quality images of fluorescently tagged proteins obtained under uniform conditions. Our OpenCell^10^ dataset of live-cell confocal images of 1,311 endogenously tagged proteins (http://opencell.czbiohub.org) meets this purpose. We reasoned that providing a fiducial channel could provide a useful reference frame for our model to capture protein localization. Hence, in addition to imaging the endogenous tag (split mNeonGreen2), we also imaged a nuclear fiducial marker (Hoechst 33342) and converted it into a distance map (Methods). On average, we imaged the localization of a given protein in 18.59 fields of view (FOV). Approximately 45 cropped images from each FOV containing 1–3 cells were then extracted for a total of 800 cropped images per protein. This scale, as well as the uniform conditions under which the images were collected, were important because our model must learn to ignore irrelevant image variance and instead focus on protein localization. Finally, in our approach all images that represent the same protein were labeled by the same unique identifier (we used the corresponding synthetic cell line identifier, but the identifier may be arbitrary). This identifier does not carry any explicit localization information, nor is it linked to any metadata or annotations, but rather is used to link together all the different images of the same protein.

### A deep-learning model to generate image representations

**Fig. 1:**
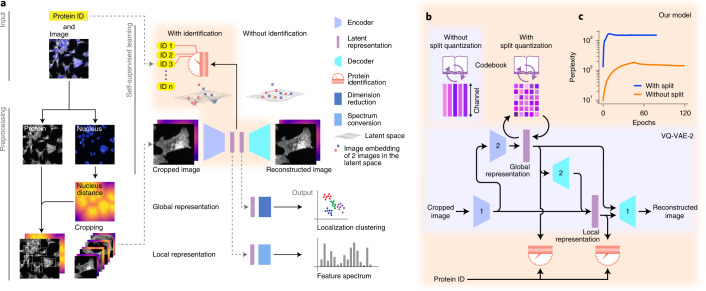
Self-supervised deep learning of protein subcellular localization with cytoself. **a**, Workflow of the learning process. Only images and the proteins identifiers are required as input. We trained our model with a second fiducial channel for the cell nuclei, but its presence is optional as its performance contribution is negligible (Fig. 4). The protein identification pretext task ensures that images corresponding to the same or similar proteins have similar representations. **b**, Architecture of our VQ-VAE-2 (ref. ^37^) -based deep-learning model featuring our two innovations: split-quantization and protein identification pretext task. Numbers in the encoders and decoders indicate encoder1, encoder2, decoder1 or decoder2 (Supplementary File 1). Global representation and local representation use different codebooks. **c**, The level of use of the codebook (that is, perplexity) increases and then saturates during training and is enhanced by applying split quantization.

Our deep-learning model is based on the vector quantized variational autoencoder architecture (VQ-VAE^35,36^). In a classical VQ-VAE, images are encoded into a quantized latent representation, a vector, and then decoded to reconstruct the input image (Fig. 1 and Supplementary File 1). The encoder and decoder are trained so as to minimize distortion between input and output images. The representation produced by the encoder is assembled by arraying a finite number of symbols (indices) that stand for vectors in a codebook (Fig. 1b and Supplementary Fig. 1). The codebook vectors themselves evolve during training so as to be most effective for the encoding–decoding task^35^. The latest incarnation of this architecture (VQ-VAE-2, ref. ^37^) introduces a hierarchy of representations that operate at multiple spatial scales (termed VQ1 and VQ2 in the original VQ-VAE-2 study). We chose this architecture as a starting point because of the large body of evidence that suggests that quantized architectures currently learn the best image representations^35,36^. As shown in Fig. 1b, we developed a variant that uses a split vector quantization scheme to improve quantization at large spatial scales (Methods and Supplementary Fig. 1). This new approach to vector quantization achieves better perplexity as shown in Fig. 1c, which means better codebook use.

### Protein localization encoding via self-supervision

Our model consists of two pretext tasks applied to each individual cropped image: first, it is tasked to encode and then decode the image as in the original VQ-VAE model. Second, it is tasked to predict the protein identifier associated with the image solely on the basis of the encoded representation. In other words, that second task aims to predict, for each single cropped image, which one of the 1,311 proteins in our library the image corresponds to. The first task forces our model to distill lower-dimensional representations of the images, while the second task forces these representations to be strong predictors of protein identity. This second task assumes that protein localization is the primary image information that is correlated to protein identity. Therefore, predicting the identifier associated with each image is key to encouraging our model to learn localization-specific representations. It is acceptable, and in some cases perfectly reasonable, for these tasks to fail. For example, when two proteins have identical localization, it is impossible to resolve the identity of the tagged proteins from images alone. Moreover, the autoencoder might be unable to perfectly reconstruct an image from the intermediate representation, when constrained to make that representation maximally predictive of protein identity. It follows that the real output of our model is not the reconstructed image, nor the predicted identity of the tagged protein, but instead the distilled image representations, which we refer to as ‘localization encodings’, that are obtained as a necessary byproduct of satisfying both pretext tasks. Specifically, our model encodes two representations for each image that correspond to two different spatial scales, the local and global representations, that correspond to VQ1 and VQ2, respectively. The global representation captures large-scale image structure scaled-down to a 4 × 4 pixel image with 576 features (values) per pixel. The local representation captures finer spatially resolved details (25 × 25 pixel image with 64 features per pixel). We use the global representations to perform localization clustering, and the local representations to provide a finer and spatially resolved decomposition of protein localization.

### Mapping the protein localization landscape with cytoself

**Fig. 2:**
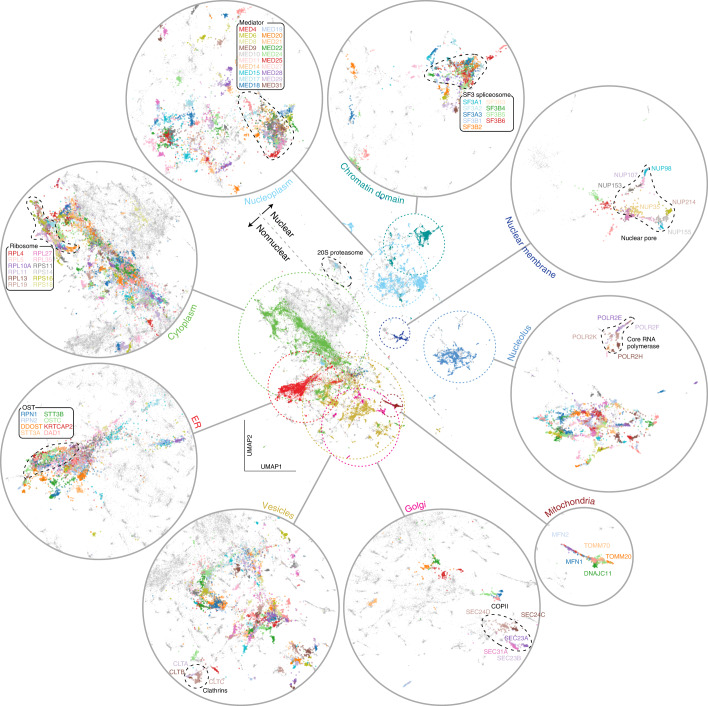
High-resolution protein localization atlas. Each point corresponds to a single image from our test dataset of 109,751 images. To reveal the underlying structure of our map, each point in the central UMAP is colored according to 11 distinct protein localization categories (mitochondria, vesicles, nucleoplasm, cytoplasm, nuclear membrane, ER, nucleolus, Golgi, chromatin domain). These categories are expanded in the surrounding circles. Tight clusters corresponding to functionally defined protein complexes can be identified within each localization category. Only proteins with a clear and exclusive localization pattern are colored, gray points correspond to proteins with other or mixed localizations. Within each localization category, the resolution of cytoself representations is further illustrated by labeling the images corresponding to individual proteins in different colors (dashed circular inserts). Note that while the colors in the central UMAP represent different cellular territories, colors in the inserts are only used to delineate individual proteins, and do not correspond to the colors used in the main UMAP. The list of annotated proteins and the subunits of each complex are indicated in Supplementary Files 2 and 5, respectively.

**Fig. 3:**
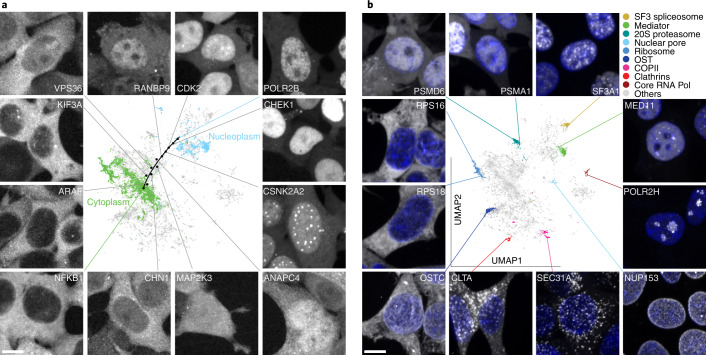
Exploring the protein localization atlas. **a**, Representative images of proteins localized along an exemplary path across the nuclear-cytoplasmic transition and over the ‘gray’ space of mixed localizations. **b**, The subunits of well-known and stable protein complexes tightly cluster together. Moreover, the complexes themselves are placed in their correct cellular contexts. Different proteins have different expression levels, hence we adjusted the brightness of each panel so as to make all localizations present in each image more visible (only minimum–maximum intensities are adjusted, no gamma adjustment used). All representative images were randomly selected. Protein localization is displayed in grayscale in both panels, the nuclei in **b** are displayed in blue. The list of the subunits of each complex are indicated in Supplementary File 5. Scale bars, 10 μm.

Obtaining image representations that are highly correlated with protein localization and invariant to other sources of heterogeneity (that is, cell state, density and shape) is only the first step for biological interpretation. Indeed, while these representations are lower dimensional than the images themselves, they still have too many dimensions for direct inspection and visualization. Therefore, we performed dimensionality reduction using the uniform manifold approximation and projection (UMAP) algorithm on the set of global localization encodings (that is, global representation in the Fig. 1) obtained from all images (Methods). In the resulting UMAP (Fig. 2) each point represents a single (cropped) image in our test dataset (that is, 10% of entire dataset, Methods), which collectively form a highly detailed map representing the full diversity of protein subcellular localizations. This protein localization atlas reveals an organization of clusters and subclusters reflective of eukaryotic subcellular architecture. We can evaluate and explore this map by labeling each protein according to its known subcellular localization obtained from independent manual annotations of our image dataset (Supplementary File 2). The most pronounced delineation corresponds to nuclear (top right) versus nonnuclear (bottom left) localizations (encircled and expanded in Fig. 2, top right and bottom left, respectively). Within the nuclear cluster, subclusters are resolved that correspond to nucleoplasm, chromatin, nuclear membrane and the nucleolus. Within each region, tight clusters that correspond to specific cellular functions can be resolved (dashed outlines). For example, subunits involved in splicing (SF3 splicesome), transcription (core RNA polymerase) or nuclear import (nuclear pore) cluster tightly together (outlined in Fig. 2, dashed outlines). Similarly, subdomains emerge within the nonnuclear cluster, the largest corresponding to cytoplasmic and vesicular localizations. Within these domains are several well delineated clusters corresponding to mitochondria, endoplasmic reticulum (ER) exit sites (COPII), ribosomes and clathrin coated vesicles (Fig. 2). The large set of unlabeled points in Fig. 2 (gray dots) correspond mainly to proteins that exhibit mixed localization patterns. Prominent among these is a band of proteins interspersed between the nuclear and nonnuclear regions (expanded in Fig. 3a). Representative proteins chosen along that path show a continuous gradation from mostly cytoplasmic to mostly nuclear localization.

### Quantifying cytoself’s clustering performance

**Fig. 4:**
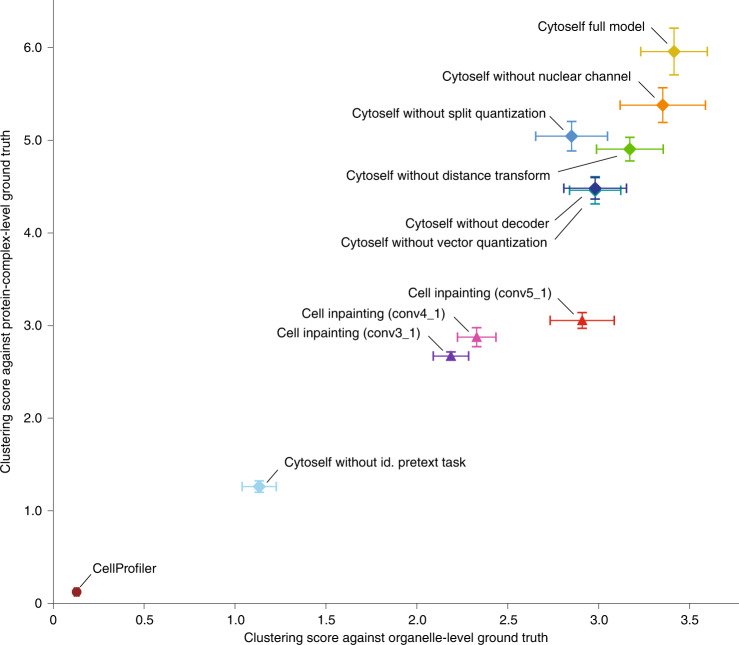
Clustering performance comparison. For each model variation, we trained five model instances, compute UMAPs for ten random seeds, compute clustering scores using organelle- and protein-complex-level ground truth and then report the mean and standard error of the mean.

**Extended Data Fig. 1:**
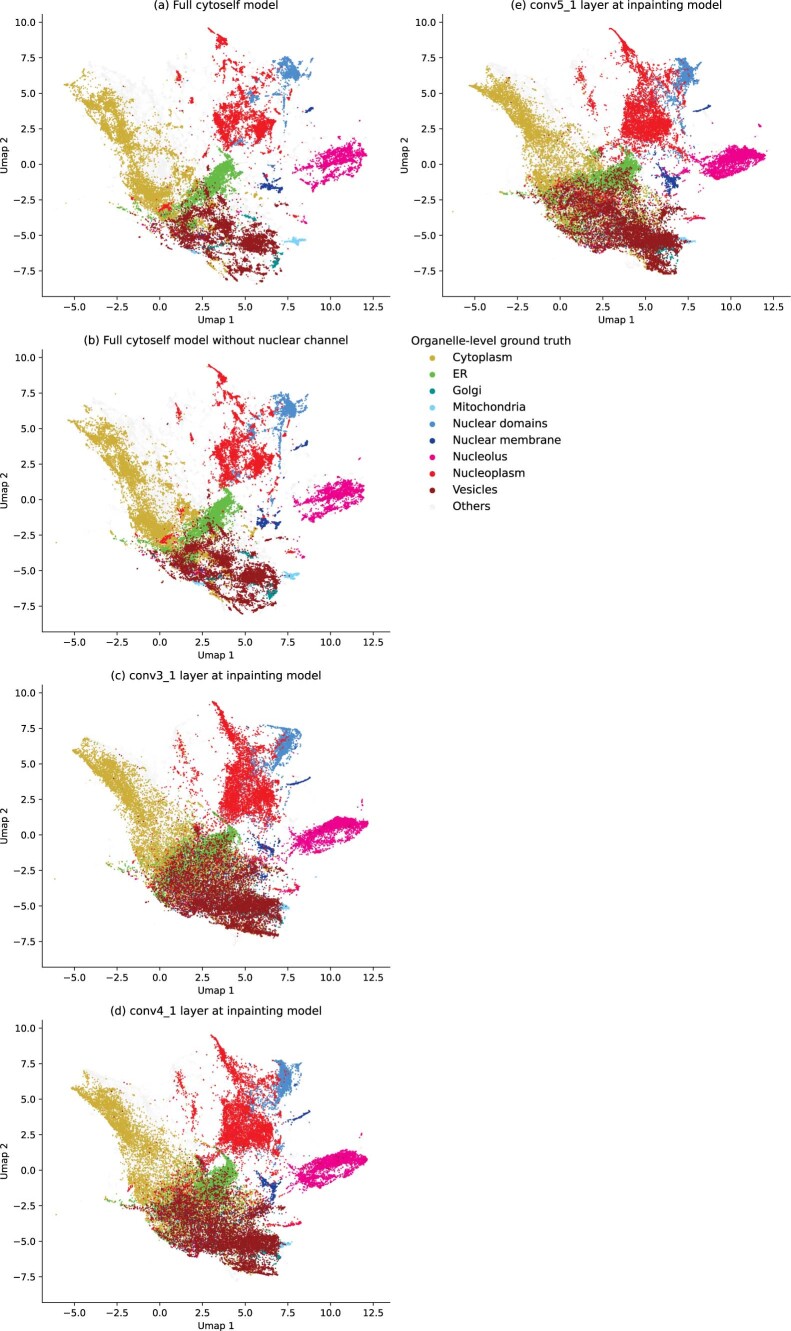
Comparing the UMAP representations between *cytoself* and cell-inpainting annotated with organelle-level ground truth. Aligned UMAPs are given to aid visual comparison.

**Extended Data Fig. 2:**
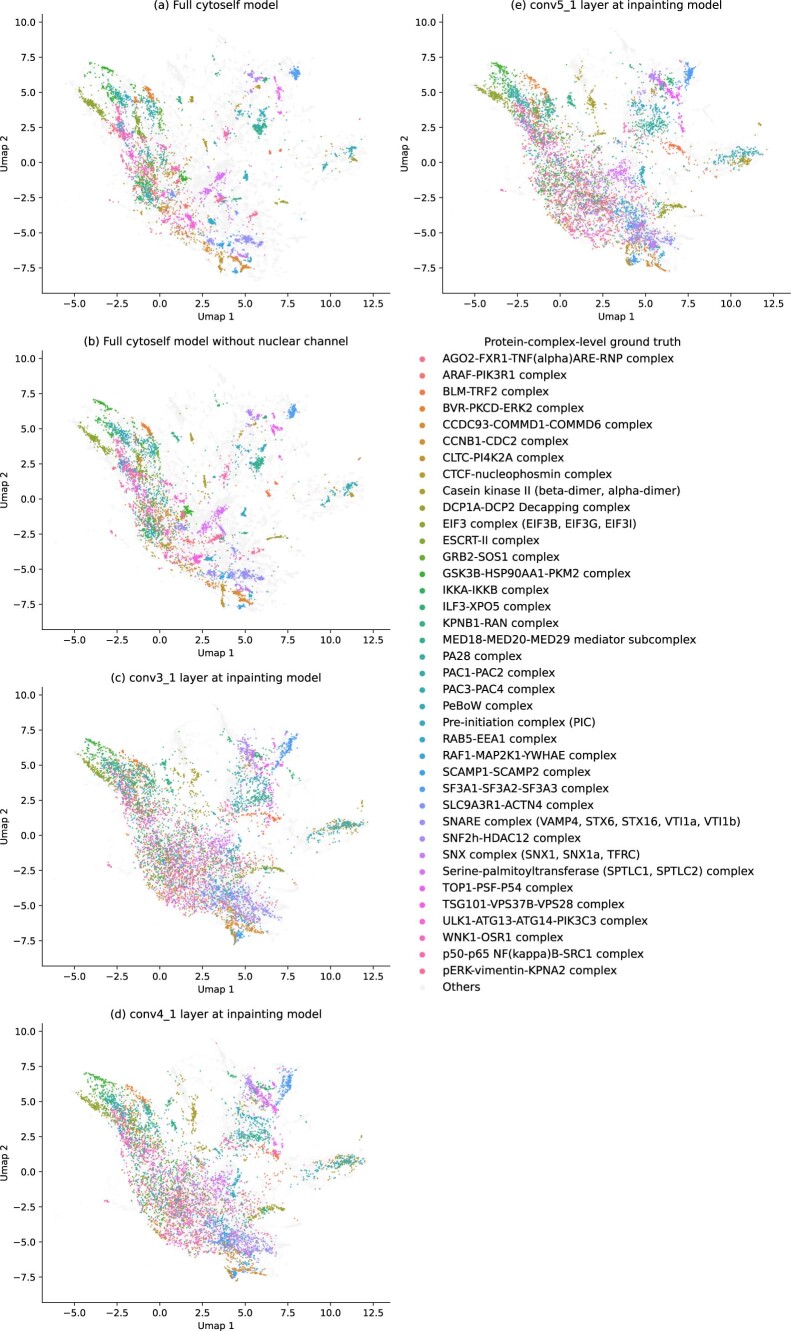
Comparing the UMAP representations between *cytoself* and cell-inpainting annotated with protein-complex-level ground truth. Aligned UMAPs are given to aid visual comparison.

To validate our results, clustering scores were computed (Methods, Fig. 4 and Table 1) using two ground-truth annotation datasets to capture known protein localization at two different scales: the first is a manually curated list of proteins with unique organelle-level localizations (Supplementary File 2), whereas the second is a list of proteins participating in stable protein complexes derived from the CORUM database^38^ (Supplementary File 3). While the first ground-truth dataset helps us assess how well our encodings cluster together proteins belonging to the same organelles, the second helps us assess whether proteins interacting within the same complex—and thus functionally related—are in proximity. We compared cytoself to other previously developed unsupervised (CellProfiler^14^) or self-supervised (Cell inpainting^11^) approaches for image featurization. We applied these methods to the OpenCell image dataset and then compared the results to that obtained by cytoself. UMAPs were calculated for each model (Methods) and compared with our set of ground-truth organelles and protein complexes. As can be seen in Extended Data Figs. 1 and 2 and Supplementary Fig. 2, the resolution obtained by cytoself exceeded that of both previous approaches. This was also apparent in our calculations of clustering scores (Fig. 4 and Table 1).

**Table 1:** Clustering performance comparison

Approach	Organelle level	Complex level
Cytoself full model	3.41 ± 0.18	5.96 ± 0.25
Without nuclear channel	3.35 ± 0.23	5.38 ± 0.19
Without distance transform	3.17 ± 0.18	4.90 ± 0.13
Without vector quantization	2.98 ± 0.14	4.46 ± 0.15
Without id. pretext task	1.13 ± 0.094	1.26 ± 0.062
Without split quantization	2.85 ± 0.20	5.04 ± 0.16
Without decoder	2.98 ± 0.17	4.48 ± 0.12
Lu et al. (conv3_1)	2.19 ± 0.097	2.67 ± 0.045
Lu et al. (conv4_1)	2.33 ± 0.11	2.88 ± 0.10
Lu et al. (conv5_1)	2.91 ± 0.18	3.06 ± 0.084
CellProfiler	0.129 ± 0.013	0.124 ± 0.0074

Our full model surpasses all variants considered, the previously reported cell-inpainting model^11^ and CellProfiler derived representations^14^. We trained the models five times, computed ten different UMAPs, computed clustering scores using organelle- and protein-complex-level ground truth, and then report the mean and standard error of the mean (mean ± s.e.m.). For the latent representations in the inpainting model, we examined the three network layers discussed in Lu et al. to produce image representations for UMAP. Note that our approach works with a single fluorescence channel whereas the approach by Lu et al. needs at least two channels.

### Identifying cytoself’s essential components

**Extended Data Fig. 3:**
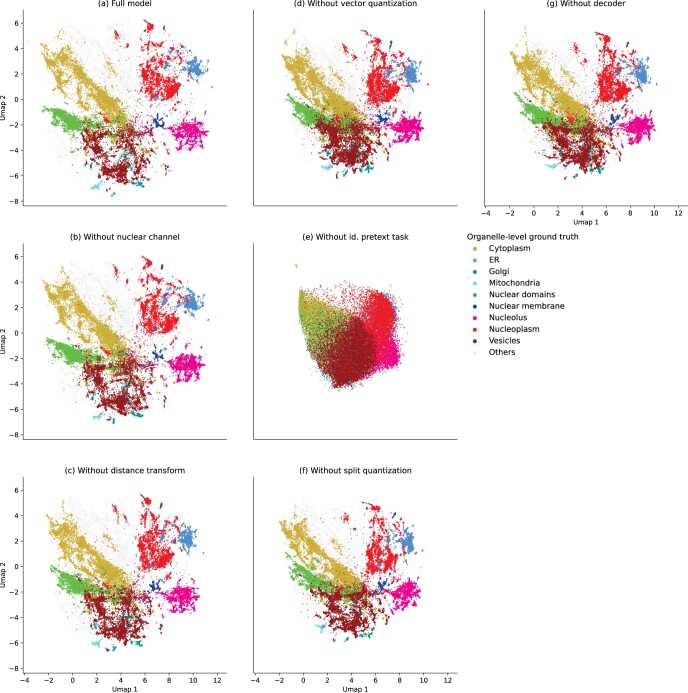
Identifying the essential components of our model with organelle-level ground truth. Protein localization UMAPs are derived after removing each components of our model separately. Aligned UMAPs are given to aid visual comparison.

**Extended Data Fig. 4:**
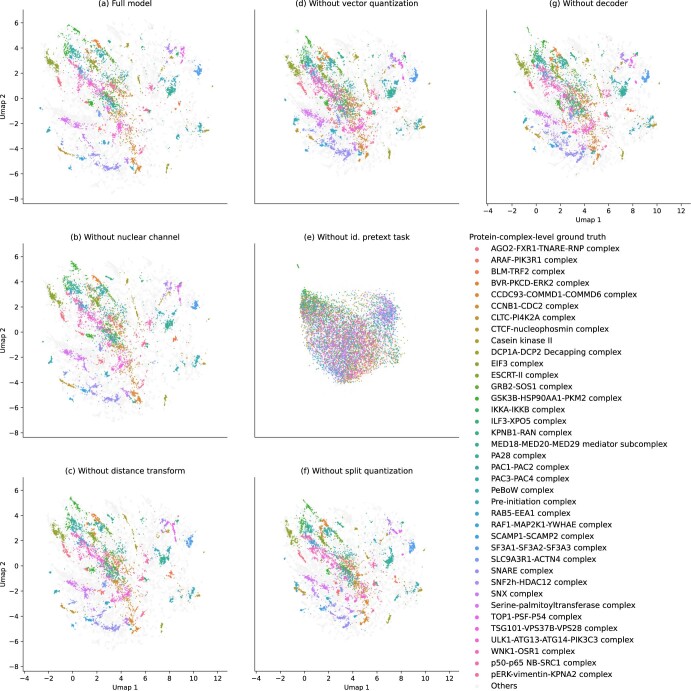
Identifying the essential components of our model with protein-complex-level ground truth. Protein localization UMAPs are derived after removing each components of our model separately. Aligned UMAPs are given to aid visual comparison.

To evaluate the impact of different aspects of our model on its clustering performance, we conducted an ablation study. We retrained our model and recomputed protein localization UMAPs after individually removing each component or input of our model (Extended Data Figs. 3 and 4), including: (1) the nuclear fiducial channel, (2) the distance transform applied to nuclear fiducial channel, (3) the split vector quantization and (4) the identification pretext task. We also quantitatively evaluated the effects of their ablation by computing clustering scores for different variants (Fig. 4 and Table 1). The UMAP results and scores from both sets of ground-truth labels make it clear that the single most important component of cytoself, in terms of clustering performance, is the protein identification pretext task. The remaining components—the nuclear channel, split quantization, vector quantization and so on—are important but not crucial. Forgoing the fiducial nuclear channel entirely led to the smallest decrease in clustering score, suggesting that our approach works well even in the absence of any fiducial marker—a notable advantage that widens the applicability of our approach and greatly simplifies the experimental design^39^. Overall, our data show a robust fit with ground truth. In conclusion, although all features contribute to the overall performance of our model, the identification pretext task is the key and necessary ingredient.

### Revealing unannotated protein localization

**Extended Data Fig. 5:**
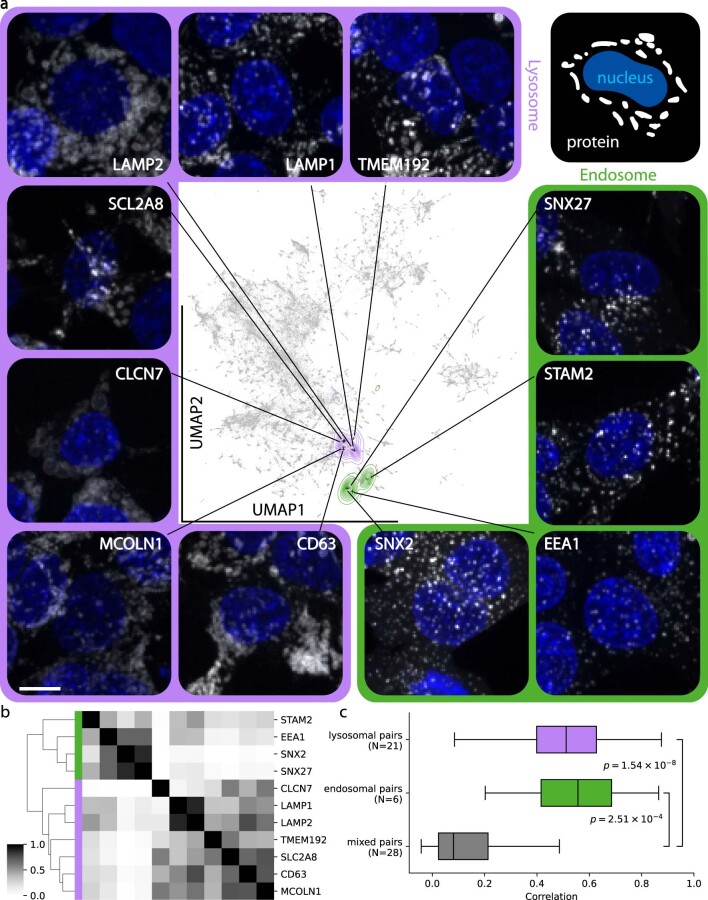
cytoself discriminates between lysosomal and endosomal proteins. **(a)** We selected 11 proteins in OpenCell annotated in Uniprot as lysosomal or endosomal that are independently confirmed as such by mass spectrometry^41,42^. We show that *cytoself* is able to distinguish the lysosomal from endosomal proteins solely on the basis of the fluorescence images. All of these proteins are annotated on the basis of the images as ‘vesicles’ in both HPA and OpenCell. The min and max intensities of each image are adjusted to ensure comparable visibility. All representative images were randomly selected from each protein. Scale bar: 10 *μm*. **(b)** Clustering of these proteins on the basis of the feature spectra. **(c)** Feature spectra correlations for pairs of lysosomal and endosomal proteins, and for mixed lysosomal-endosomal pairs. Each box indicates the extent from the first to the third quartile of the data, with a line representing the median. The whiskers indicates 1.5 times the inter-quartile range. The p-values are computed using two-sided Mann–Whitney U test.

The key advantage of self-supervised approaches is that they are not limited by the quality, completeness or granularity of human annotations. To demonstrate this, we asked whether cytoself could resolve subtle localization differences that are not present in image-derived manual annotations: focusing on proteins localized to intracellular vesicles. Even though several known subcategories of vesicles exist (for example, lysosomes versus endosomes), in both OpenCell and Human Protein Atlas annotations, these groups are annotated simply as ‘vesicles’. This reflects the difficulty for human curators to accurately distinguish and classify localization subcategories that present similarly in the images. To test whether our self-supervised approach manages to capture these subcategories, we focused on a curated list of endosomal as well as lysosomal proteins identified by an objective criterion. Specifically, we selected proteins annotated as lysosomal (GO 000576500) or endosomal (GO 0031901) in Uniprot^40^ (excluding targets annotated to reside in both compartments), and for which localization in each compartment has been confirmed independently by mass spectrometry^41,42^. As shown in Extended Data Fig. 5, the representation of the lysosomal versus endosomal images derived from cytoself form two distinct, well-separated clusters (*P* < 10^−3^, Mann–Whitney *U-*test). This demonstrates that self-supervised approaches are not limited by ground-truth annotations and can reveal subtle differences in protein localization not explicitly present in existing databases.

### Extracting feature spectra for quantitative analysis

**Fig. 5:**
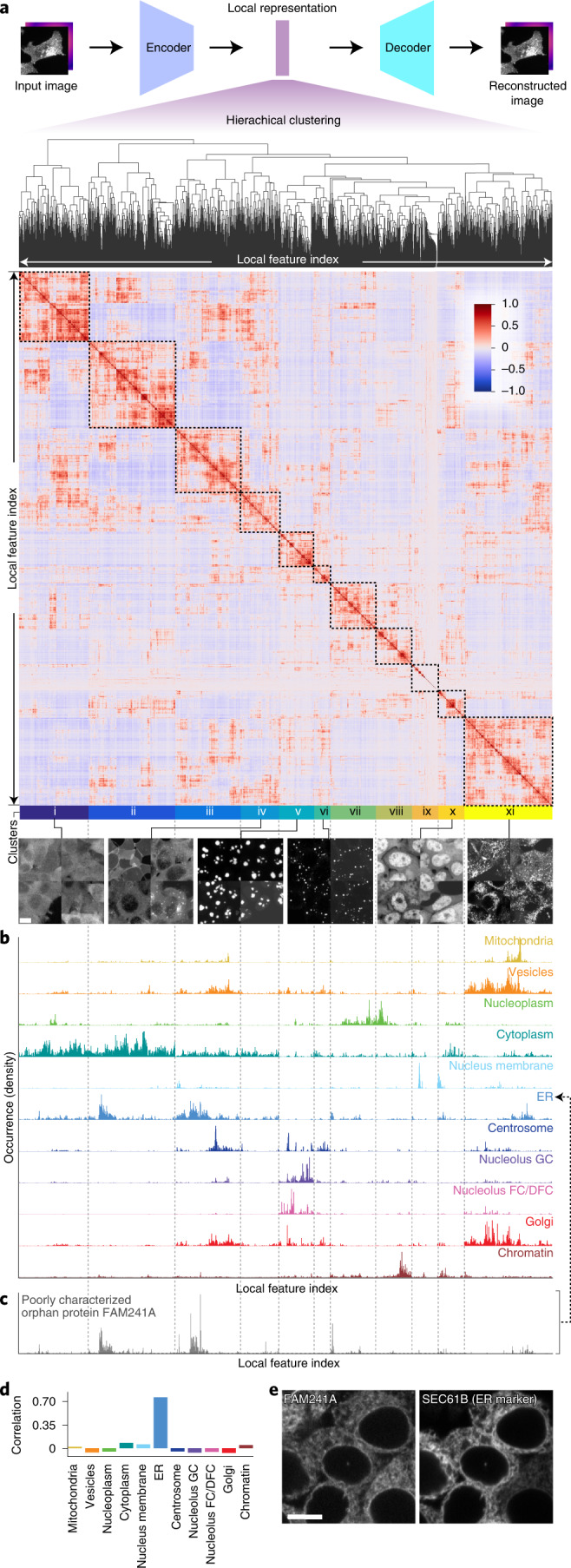
Feature spectral analysis. **a**, Features in the local representation are reordered by hierarchical clustering to form a feature spectra (Extended Data Fig. 6). The color bar indicates the strength of correlation. Negative values indicate anti-correlation. On the basis of the feature clustering, we manually identified 11 primary top-level clusters, which are illustrated with representative images (Supplementary Fig. 3). Those images have the highest occurrence of the corresponding features. **b**, Average feature spectrum for each unique localization family. Occurrence indicates how many times a quantized vector is found in the local representation of an image. All spectra, as well as the heatmap, are vertically aligned. **c**, The feature spectrum of FAM241A, a poorly characterized orphan protein. **d**, Correlation between FAM241A and other unique localization categories. The highest correlation is 0.777 with ER, next is 0.08 with cytoplasm. **e**, Experimental confirmation of the ER localization of FAM241A. The localization of FAM241A to the ER is experimentally confirmed by coexpression of a classical ER marker (mCherry fused to the SEC61B transmembrane domain, left) in FAM241A-mNeonGreen endogenously tagged cells (right). The ER marker is expressed using transient transfection. As a consequence, not all cells are transfected and levels of expression may vary. Scale bars, 10 μm.

**Extended Data Fig. 6:**
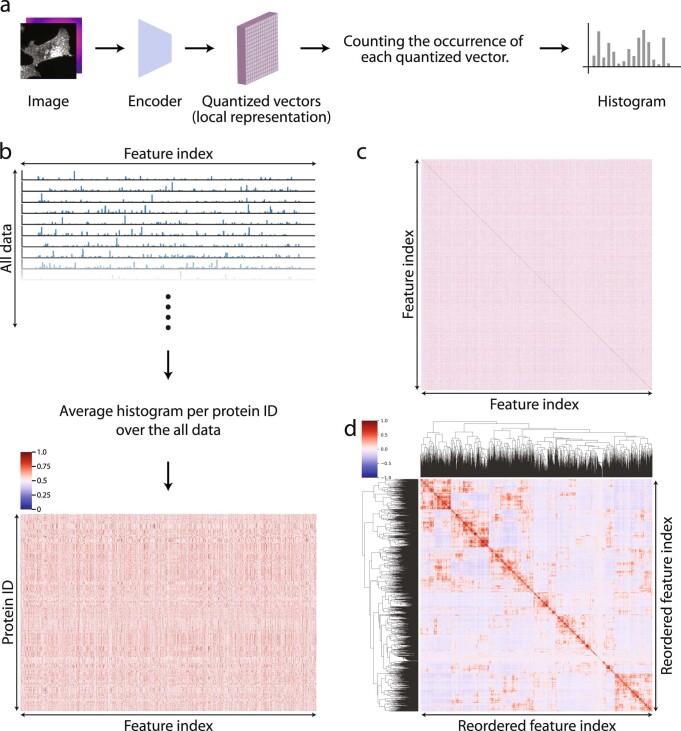
Process of constructing feature spectra. **(a)** First, the quantized vectors in the local representation were extracted and converted to a histogram by counting the occurrence of each quantized vector. **(b)** Next, taking the average of the histograms per protein ID over all the data to create a 2D histogram. **(c)** Pearson’s correlations between any two representation indices were calculated and plotted as a 2D matrix. **(d)** Finally, hierarchical clustering was performed on the correlation map so that similar features are clustered together, revealing the structure inside the local representation. The whole process corresponds to the Spectrum Conversion in Fig. 1a.

Cytoself can generate a highly resolved map of protein localization on the basis of distilled image representations. Can we dissect and understand the features that make up these representations and interpret their meaning? To identify and better define the features that make up these representations, we created a feature spectrum of the main components contributing to each protein’s localization encoding. The spectra were constructed by calculating the histogram of codebook feature indices from the local representations in Fig. 1 (see Extended Data Fig. 6 and Methods for details). To group related and possibly redundant features together, we performed hierarchical biclustering^43^ (Fig. 5a), and thus obtained a meaningful linear ordering of features by which the spectra can be sorted. This analysis reveals feature clusters of which we manually selected 11 from the top levels of the feature hierarchy (Fig. 5a, bottom and Supplementary Fig. 3).

Representative images from each cluster illustrate the variety of distinctive localization patterns that are present at different levels across all proteins. For example, the features in the first clusters (i, ii, iii and iv) correspond to a wide range of diffuse cytoplasmic localizations. Cluster v features are unique to nucleolar proteins. Features making up cluster vi correspond to very small and bright punctate structures that are often characteristic of centrosomes, vesicles or cytoplasmic condensates. Clusters vii, viii and x correspond to different types of nuclear localization pattern. Cluster ix are dark features corresponding to nonfluorescent background regions. Finally, cluster xi corresponds to a large variety of more abundant, punctate structures occurring throughout the cells, primarily vesicular, but also Golgi, mitochondria, cytoskeleton and subdomains of the ER. For a quantitative evaluation, we computed the average feature spectrum for all proteins belonging to each localization category present in our reference set of manual annotations (for example, Golgi, nucleolus and so on; Fig. 5b and Supplementary File 4). This analysis confirms that certain spectral clusters are specific to certain localization categories and thus correspond to characteristic textures and patterns in the images. For example, the highly specific chromatin and mitochondrial localizations both appear to elicit very narrow responses in their feature spectra.

### Predicting protein organelle localization with cytoself

**Extended Data Fig. 7:**
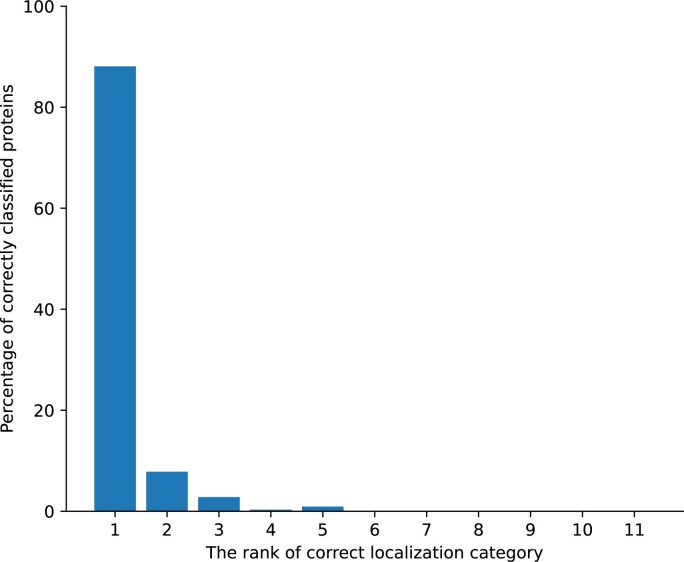
Predicting the localization category of mono-localized OpenCell proteins by correlating the *cytoself* spectra of each protein with the representative spectra of each category – in a leave-one-out fashion. Result: 88% of proteins are correctly classified. For 96% of proteins the correct annotation is within the top 2 predictions, and for 99% it is within the top 3 predictions.

We next asked whether feature spectra could be used to predict the localizations of proteins not present in our training data. For this purpose, we computed the feature spectrum of FAM241A: a protein of unknown function that was not present in the training dataset. Its spectrum is most correlated to the consensus spectrum of proteins belonging to the ER (Fig. 5b–d and Supplementary Fig. 4). Indeed, FAM241A’s localization to the ER was validated experimentally by coexpression experiments showing that endogenously tagged FAM241A colocalizes with an ER marker (Fig. 5e). In a companion study^10^, we further validated by mass spectrometry that FAM241A is in fact a new subunit of the oligosaccharyltransferase complex, responsible for cotranslational glycosylation at the ER membrane. Our successful prediction of the localization of FAM241A suggests that cytoself encodings can be used more generally to predict organelle-level localization categories. To demonstrate this, we focused on proteins annotated to localize to a single organelle (that is, not multi-localizing, Supplementary File 4). For each of these proteins, we recomputed the representative spectra for each of their known localization categories (that is, ER, mitochondria, Golgi and so on), but leaving out that protein, and then applied the same spectral correlation as described for FAM241A. This allows us to predict the protein’s localization by identifying the organelle with which its spectrum correlates best. Extended Data Fig. 7 shows the accuracy of the predictions derived from this approach: for 88% of proteins, the spectra correlate best with the correctly annotated organelle. For 96% of proteins, the correct annotation is within the top two predictions and for 99% it is within the top three predictions. Overall, this form of cross-validation verifies the discriminating power of our spectra and shows that the information encoded in each protein’s spectrum can be interpreted to predict subcellular localization.

### Cytoself applicability beyond OpenCell data

**Extended Data Fig. 8:**
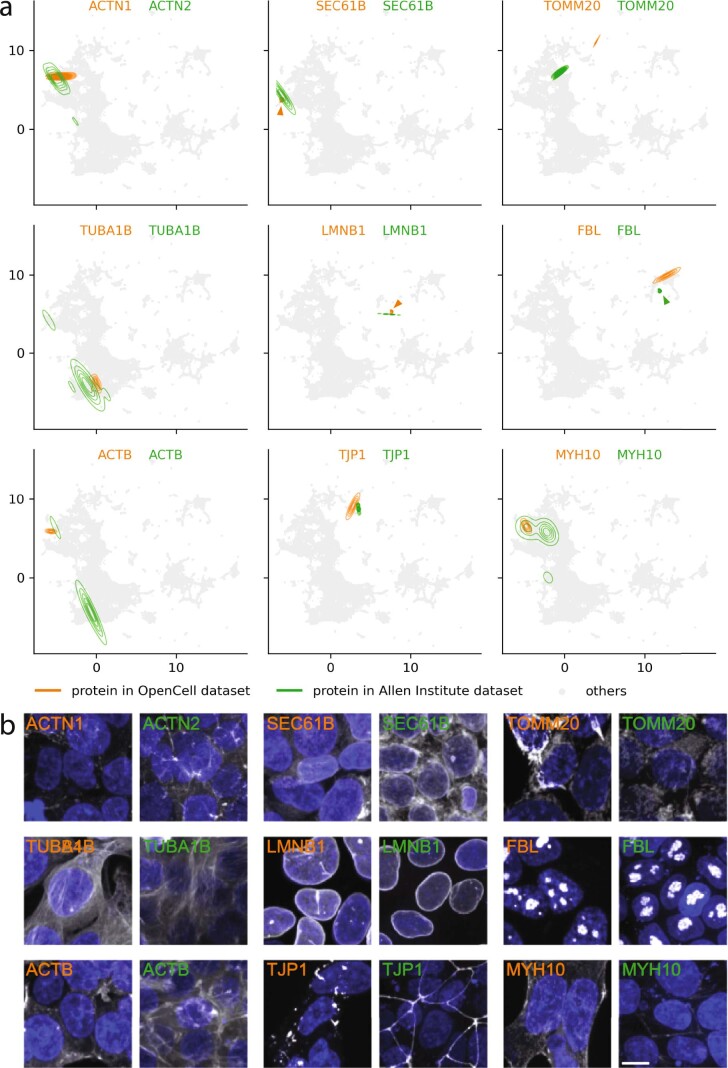
Visualizing the predicted localization categories of proteins present both in OpenCell and the Allen Institute dataset. The *cytoself* model is trained only on OpenCell data which is the same *full* model used throughout this work. UMAPs **(a)** and example images **(b)** from OpenCell and Allen Institute datasets for the same or related proteins. The min and max intensities of each image are adjusted to ensure comparable visibility. All representative images were randomly selected. Scale bar 10 *μm*. Protein names and contour lines in orange color are from OpenCell dataset, and those in green color are from Allen Institute dataset.

Can cytoself make reasonable protein localization predictions for images from datasets other than OpenCell? To answer this question, we chose data from the Allen Institute Cell collection^44^, which also uses endogenous tagging and live-cell imaging, making their image data directly comparable to ours. The Allen collection uses a cell line (WTC11, induced pluripotent stem cell) whose overall morphology is very different from the cell line used for OpenCell (human embryonic kidney 293T: HEK293T). We reasoned that if cytoself manages to capture true features of protein localization, a compelling validation would be that its performance would be cell-type agnostic. Indeed, localization encodings for images from the Allen dataset generated by a cytoself model trained only on OpenCell images revealed a strong concordance between the embeddings of the same (or closely related) protein that were imaged in both cell datasets (Extended Data Fig. 8a). This shows that our model manages to predict protein localization even under conditions that were not directly included for training. To facilitate comparison, we focused on the intersection set of nine proteins found in both the OpenCell and Allen datasets (Extended Data Fig. 8b). We ran the same organelle localization prediction task and observed that in 88% (eight out of nine) of cases the correct localization is among the top three predictions (Supplementary Fig. 5).

### Hypothesizing protein-complex membership from images

The resolving power of our approach is further illustrated by examining known stable protein complexes, which are found to form well delineated clusters in our localization UMAP (see examples highlighted in Fig. 2, dashed line). Fluorescent images of 11 representative subunits from these complexes illustrate these discrete localization patterns (Fig. 3b). To substantiate these observations quantitatively, we computed the correlation of feature spectra between any two pairs of proteins in our dataset. This showed a significantly higher correlation for protein pairs annotated to belong to the same complex in CORUM compared to pairs that are not (*P* < 10^−10^, Mann–Whitney *U*-test; Supplementary Fig. 6a). To further evaluate the relationship between proximity in feature space and protein-complex membership, we examine the proportion of proteins in OpenCell that share complex membership with their most-correlated neighboring protein (Supplementary Fig. 6b). We find that 83% of highly correlated (>0.95) neighbor proteins are in the same complex, and even more weakly correlated (>0.8) proteins are localized to complexes 60% of the time. These results confirm that close proximity in feature space is highly indicative of protein-complex membership and suggests that the features derived by cytoself contain fine-grained information related to very specific functional relationships.

## Discussion

We have shown that a self-supervised training scheme can produce image representations that capture the organization of protein subcellular localization (Fig. 2), solely on the basis of a large high-quality dataset of fluorescence images. Our model generates a high-resolution localization atlas capable of delineating not only organelles, but also protein complexes. Moreover, we can represent each image with a feature spectrum to better analyze the repertoire of localization patterns present in our data. Since a protein’s localization is highly correlated with its cellular function, cytoself will be an invaluable tool to make preliminary functional predictions for unknown or poorly studied proteins, and for quantitatively studying the effect of cellular perturbations and cell state changes on protein subcellular localization.

Our method makes few assumptions, but imposes two pretext tasks (that is, image and protein identity). Of these, requiring the model to identify proteins based solely on their localization encodings was essential. We also included Hoechst DNA-staining as a fiducial marker, assuming that this would provide a spatial reference frame against which to interpret localization. However, this added little to the performance of our model in terms of clustering score. By comparison, the self-supervised approach by Lu et al.^11^ applied a pretext task that predicts the fluorescence signal of a labeled protein in one cell from its fiducial markers and from the fluorescence signal in a second, different cell from the same FOV. This assumes that fiducial channels are available, and that protein fluorescence is always well-correlated to these fiducials. In contrast, our approach only requires a single fluorescence channel and yields better clustering performance (Fig. 4 and Table 1).

The main difference between our work and the problem addressed by the Human Protein Atlas Image Classification competition^23^ is that we do not aim to predict localization patterns on the basis of manual annotations. Instead, we aim to discover de novo the landscape of possible protein localizations. This frees us from the limitations of these annotations that include: lack of uniform coverage, uneven annotation granularity, human perceptive biases and existing preconceptions on the architecture of the cell. This also circumvents the time-intensive efforts required to manually annotate images.

While powerful, there remain a few avenues for further development of cytoself. For example, we trained our model using two-dimensional (2D) maximum-intensity *z*-projections and have not yet leveraged the full three-dimensional (3D) confocal images available in the OpenCell^10^ dataset. The third dimension might confer an advantage for specific protein localization patterns that are characterized by specific variations along the basal-apical cell axis. Other important topics to explore are the automatic suppression of residual batch effects, improved cell segmentation via additional fiducial channels, use of label-free imaging modalities, as well as automatic rejection of anomalous or uncharacteristic cells from our training dataset. More fundamentally, notable conceptual improvements will require an improved self-supervised model that explicitly disentangles cellular heterogeneity from localization diversity^45^.

More generally, our ability to generate data is outpacing the human ability to manually annotate it. Moreover, there is already ample evidence that abundance of image data has a quality all its own: Increasing the size of an image dataset often has a higher impact on performance than improving the algorithm itself^46^. We envision that self-supervision will be a powerful tool to handle the large amount of data produced by new instruments, end-to-end automation and high-throughput image-based assays.

## Methods

### Fluorescence image dataset

All experimental and imaging details can be found in our companion study^10^. Briefly, HEK293T cells were genetically tagged with split-fluorescent proteins using CRISPR-based techniques^47^. After nuclear staining with Hoechst 33342, live cells were imaged with a spinning-disk confocal microscope (Andor Dragonfly). Typically, 18 FOV were acquired for each one of the 1,311 tagged proteins, for a total of 24,382 three-dimensional images of dimension 1,024 × 1,024 × 22 voxels.

### Image data preprocessing

Each 3D confocal image was first reduced to two dimensions using a maximum-intensity projection along the *z* axis followed by downsampling in the *xy* dimensions by a factor of two to obtain a single 2D image per FOV (512 × 512 pixels). To help our model make use of the nuclear fiducial label, we applied a distance transform to a nucleus segmentation mask (below). The distance transform is constructed so that pixels within the nucleus were assigned a positive value that represents the shortest distance from the pixel to the nuclear boundary, and pixel values outside the nucleus were assigned a negative value that represents the shortest distance to the nuclear boundary (Fig. 1a). For each dual-channel and full FOV image, multiple regions of dimension 100 × 100 pixels were computationally chosen so that at least one cell is present and centered, resulting in a total of 1,100,253 cropped images. Cells (and their nuclei) that are too close to image edges are ignored. The raw pixel intensities in the fluorescence channel are normalized between 0 and 1, and the nuclear distance channel is normalized between −1 and 1.

### Nucleus segmentation

Nuclei are segmented by first thresholding the nucleus channel (Hoechst staining) and then applying a custom algorithm to segment any under-segmented nuclei. In the thresholding step, a background mask is generated by applying a low-pass Gaussian filter to the raw image, then thresholding it using a threshold value calculated by the iterative Minimum Cross Entropy method^48,49^. Under-segmented nuclei in the resulting mask are then segmented by applying the following steps: (1) we generate a second mask by applying a Laplacian of the Gaussian (LoG) filter to the original image, thresholding it at zero, and multiplying it by the background mask from the intensity thresholding step, (2) we morphologically close this second mask and fill holes to eliminate intra-nuclear holes or gaps (empirically, this requires a closing disk of radius at least 4 pixels), (3) we multiply the second mask again by the background mask to restore any true morphological holes that were present in the background mask, (4) we generate a mask of the local minima in the original LoG-filtered image using an empirically selected percentile threshold, and finally (5) we iterate over regions in this local-minima mask and remove them from the second mask if they partially overlap with the background of the refined mask. The second mask is then the final nucleus segmentation mask.

### Detailed model architecture

All details of our model architecture are given in Supplementary File 1 and a diagram is shown in Fig. 1b. First, the input image (100 × 100 × 2 pixels) is fed to encoder1 to produce a set of latent vectors that have two destinations: encoder2 and VQ1 VectorQuantizer layer. In the encoder2, higher level representations are distilled from these latent vectors and passed to the output. The output of encoder2 is quantized in the VQ2 VectorQuantizer layer to form what we call ‘global representation’. The global representation is then passed to the fc2 classifier for purposes of the classification pretext task. It is also passed on to decoder2 to reconstruct the input data of encoder2. In this way, encoder2 and decoder2 form an independent autoencoder. The function of layer mselyr1 is to adapt the output of decoder2 to match the dimensions of the output of encoder1, which is identical to the dimensions of the input of encoder2. In the case of the VQ1 VectorQuantizer layer, vectors are quantized to form what we call the local representations. The local representation is then passed to the fc1 classifier for purposes of the classification pretext task, as well as concatenated to the global representation that is resized to match the local representations’ dimensions. The concatenated result is then passed to the decoder1 to reconstruct the input image. Here, encoder1 and decoder1 form another autoencoder.

### Split quantization

In the case of our global representation, we observed that the high level of spatial pooling required (4 × 4 pixels) led to codebook under-use because the quantized vectors are too few and each one of them has too many dimensions (Fig. 1b). To solve this challenge, we introduced the concept of split quantization. Instead of quantizing all the dimensions of a vector at once, we first split the vectors into subvectors of equal length and then quantize each subvectors using a shared codebook. The main advantage of split quantization when applied to the VQ-VAE architecture is that one may vary the degree of spatial pooling without changing the total number of quantized vectors per representation. In practice, to maintain the number of quantized vectors while increasing spatial pooling, we simply split along the channel dimension. We observed that the global representations’ perplexity, which indicates the level of use of the codebook, substantially increases when split quantization is used compared to standard quantization (Fig. 1c). As shown in Supplementary Fig. 1, split quantization is performed along the channel dimension by splitting each channel-wise vector into nine parts, and quantizing each of the resulting ‘subvectors’ against the same codebook. Split quantization is only needed for the global representation.

### Global and local representations

The dimensions of the global and local representations are 4 × 4 × 576 and 25 × 25 × 64 voxels, respectively. These two representations are quantized with two separate codebooks consisting of 2,048 64-dimensional features (or codes).

### Identification pretext task

The part of our model that is tasked with identifying a held-back protein is implemented as a two-layer perceptron built by alternatively stacking fully connected layers with 1,000 hidden units and nonlinear ReLU layers. The output of the classifier is a one-hot encoded vector for which each coordinate corresponds to one of the 1,311 proteins. We use categorical cross entropy as classification loss during training.

### Computational efficiency

Due to the large size of our image data (1,100,253 cropped images of dimensions 100 × 100 × 2 pixels) we recognized the need to make our architecture more efficient and thus allow for more design iterations. We opted to implement the encoder using principles from the EfficientNet architecture to increase computational efficiency without losing learning capacity^50^. Specifically, we split the model of EfficientNetB0 into two parts to make the two encoders in our model (Supplementary File 1). While we did not notice a loss of performance for the encoder, EfficientNet did not perform as well for decoding. Therefore, we opted to keep a standard architecture based on a stack of residual blocks for the decoder^51^

### Training protocol

The whole dataset (1,100,253 cropped images) was split into 8:1:1 into training, validation and testing data, respectively. All results shown in the figures are from testing data. We used the Adam optimizer with the initial learning rate of 0.0004. The learning rate was multiplied by 0.1 every time the validation loss did not improve for four epochs, and the training was terminated when the validation loss did not improve for more than 12 consecutive epochs. Images were augmented by random rotation and flipping in the training phase.

### Dimensionality reduction and clustering

Dimensionality reduction is performed using the UMAP^52^ algorithm. We used the reference open-source python package umap-learn (v.0.5.0) with default values for all parameters (that is, the Euclidean distance metric, 15 nearest neighbors and a minimal distance of 0.1). We used AlignedUMAP for the clustering performance evaluation to facilitate the comparison of the different projections derived from three variants of the previously described cell-inpainting model^11^ (Extended Data Figs. 1 and 2) or all seven variants of our model (Extended Data Figs. 3 and 4). Hierarchical biclustering was performed using seaborn (v.0.11.1) with its default settings.

### Ground-truth labels for localization

To evaluate the clustering performance, we used two sets of ground-truth labels at two different cellular scales: a manually curated list of proteins with exclusive organelle-level localization patterns (Supplementary File 2) and 38 protein complexes collected from the CORUM database^38^ (Supplementary File 3). The 38 protein complexes were collected based on the following conditions: (1) all subunits are present in the OpenCell data, (2) no overlapping subunit across the complexes and (3) each protein complex consists of more than one distinct subunit.

For the evaluation of feature spectra, we simply extracted the proteins with single-localization annotation based on the localization annotation given by the OpenCell database (Supplementary File 4).

### Clustering score

To calculate a clustering score, we assume a collection of *n* points (vectors) in ${{\mathbb{R}}}^{m}$, $S=\{{\bf x}_{i}\in {{\mathbb{R}}}^{m}| 0\le i\le n\}$, and that we have a (ground truth) assignment of each point **x**_*i*_ to a class *C*_*j*_, and these classes form a partition of *S*:

$$S=\mathop{\bigcup}\limits_{j}{C}_{j}$$

Ideally, the vectors **x**_*i*_ are such that all points in a class are tightly grouped together, and that the centroids of each class are as far apart from each other as possible. This intuition is captured in the following definition of our clustering score:

$${{\Gamma }}({C}_{i})=\frac{{\sigma }^{* }({\{{\mu }^{* }({C}_{j})\}}_{j})}{{\mu }^{* }({\{{\sigma }^{* }({C}_{j})\}}_{j})}$$

where {.}_*k*_ denotes the set of values obtained by evaluating the expression for each value of parameter *k*, and where μ^*^ and σ^*^ stand for the robust mean (median) and robust standard deviation (computed using medians). Variance statistics were obtained by training the model variant five times followed by computing the UMAP ten times per trained model.

### Feature spectrum

Extended Data Fig. 6a illustrates the workflow for constructing the feature spectra. Specifically, we first obtain the indices of quantized vectors in the latent representation for each image crop, and then calculate the histogram of indices in all images of each protein. As a result, we obtain a matrix of histograms in which rows correspond to protein identification (ID) and columns to the feature indices (Extended Data Fig. 6b). At this point, the order of the columns (that is, the feature indices) is arbitrary. Yet, different features might be highly correlated and thus either related or even redundant (depending on how ‘saturated’ the codebook is). To meaningfully order the feature indices, we compute the Pearson correlation coefficient between the feature index ‘profiles’ (the columns of the matrix) for each pair of feature indices to obtain a 2,048 × 2,048 pairwise correlation matrix (Extended Data Fig. 6c). Next, we perform hierarchical biclustering in which the feature indices with the most similar profiles are iteratively merged^53^. The result is that features that have similar profiles are grouped together (Extended Data Fig. 6d). This ordering yields a more meaningful and interpretable view of the whole spectrum of feature indices. We identified several clusters from the top levels of the feature hierarchy and manually segment them into 11 major feature clusters (ordered i to xi). Finally, for a given protein, we can produce a interpretable feature spectrum by ordering the horizontal axis of the quantized vectors histogram in the same way.

### Training cell-inpainting model on OpenCell data

The cell-inpainting model was constructed using the code provided by its original authors (https://github.com/alexxijielu/paired_cell_inpainting). The whole dataset was split into training, validation and testing sets (8:1:1). All results shown in the figures are computed on the basis of the test set. We used the Adam optimizer with the initial learning rate of 0.0004. The learning rate was multiplied by 0.1 every time the validation loss did not improve for four epochs, and the training was terminated when the validation loss did not improve for more than 12 consecutive epochs. The features to generate UMAP were extracted from layers denoted as ‘conv3_1’, ‘conv4_1’ and ‘conv5_1’ by the authors.

### Applying cytoself on the Allen Institute dataset

Image data from the Allen Institute were downloaded from https://www.allencell.org/data-downloading.html#DownloadImageData. Patches were made following the same procedure as for the OpenCell dataset including max-intensity projection and downsampling to match pixel resolutions. Nuclear centers were determined using the nuclear label included in the Allen Institute dataset. We randomly selected 80 patches per protein and used these for analysis.

### Feature extraction with CellProfiler

CellProfiler v.4.2.1 was used to extract features from nuclear images (without distance transform) and fluorescence protein images. In the case of cytoself, we computed all features compatible to the data including texture features up to scale 15, for a total of 1,397 features that required 2 days of computation. Only features that did not require object detection were used, including granularity, texture and the correlations between the two channels. Each feature was standardized by subtracting its mean followed by dividing by its standard deviation.

### Evaluation of feature correlation against protein complex

The Pearson correlation between any two proteins found in both the OpenCell and CORUM databases were computed with their feature spectra as the proximity metrics in the feature space. For each protein, we found the ‘nearest protein’ with which it had the highest correlation, and incremented the number if the correlation was higher than a given threshold, and if both of them shared at least one complex in the CORUM database. To take into account the strength of correlation, we varied the minimal correlation threshold thus obtaining the curve shown in Supplementary Fig. 6b.

### Statistics and reproducibility

All box plots were generated using matplotlib (v.3.4.2). Each box indicates the extent from the first to the third quartile of the data, with a line representing the median. The whiskers indicate 1.5 times the interquartile range. Scipy (v.1.8.0) was used to compute *P* values and Pearson’s correlations.

### Software and hardware

All deep-learning architectures were implemented in TensorFlow v.1.15 (ref. ^54^) on Python v.3.7. Training was performed on NVIDIA V100-32GB GPUs.

### Reporting summary

Further information on research design is available in the Nature Research Reporting Summary linked to this article.

## Online content

Any methods, additional references, Nature Research reporting summaries, source data, extended data, supplementary information, acknowledgements, peer review information; details of author contributions and competing interests; and statements of data and code availability are available at 10.1038/s41592-022-01541-z.

## Supplementary information

Supplementary Information Supplementary Text, Figs. 1–9 and Files 1–5.

Reporting Summary.

Supplementary Data **a**–**e**, Detailed structure of VQ-VAE model, including the whole model structure (**a**), the structure of encoder1 (**b**), the structure of encoder2 (**c**), the structure of decoder1 (**d**) and the structure of decoder2 (**e**).

Supplementary Table The ground truth used for evaluating clustering performance at organelle level.

Supplementary Table A list of protein subunits collected from CORUM as a ground truth to compute clustering scores. See Methods for how they were selected.

Supplementary Table The ground truth used for evaluating feature spectra.

Supplementary Table A list of protein subunits for protein complexes mentioned in Figs. 2 and 3b.

## Figures and Tables

**Extended Data Fig. 9:**
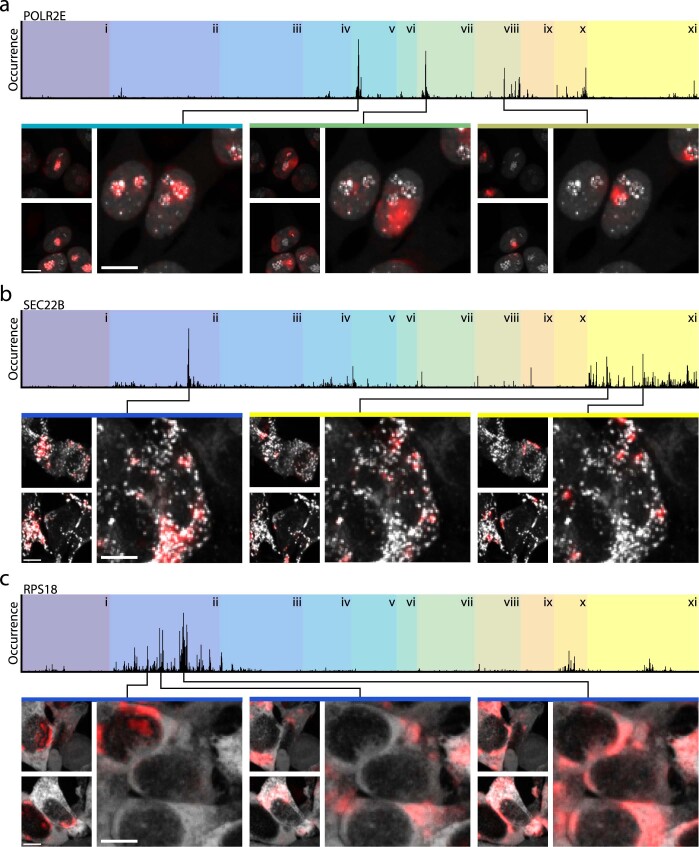
Interpreting image spectral features. Feature spectra were computed for each example proteins **(a)** POLR2E, **(b)** SEC22B, and **(c)** RPS18. Subsequently, information derived from the indicated major peaks of their feature spectra was removed by zeroing them out before passing the features again through the decoder. Highlighted in red are the differences between the resulting output images for the corresponding features and reconstructed image with full features on. The feature classes outlined in Fig. 5 are shown as background color for reference. The pixel intensities are rescaled to the minimum and maximum of each image. Scale bars: 10*μm*.

## Data Availability

The image data used in this work are available at https://github.com/royerlab/cytoself. The CORUM database is available at http://mips.helmholtz-muenchen.de/corum/. Image data from the Allen Institute are available at https://www.allencell.org/data-downloading.html#DownloadImageData.

## Extended data

are available for this paper at 10.1038/s41592-022-01541-z.

## Code availability

Source code for the models used in this work is available at https://github.com/royerlab/cytoself.
