# Supplementary material for: Self-supervised deep learning encodes high-resolution features of protein subcellular localization
Source: Nat Methods. 2022 Jul 25;19(8):995–1003. doi: 10.1038/s41592-022-01541-z (PMC9349041; doi:10.1038/s41592-022-01541-z)
Supplement: Supplementary file 1 — Supplementary Text, Figs. 1–9 and Files 1–5. [file 41592_2022_1541_MOESM1_ESM.pdf]

nature portfolio

https://doi.org/10.1038/s41592-022-01541-z

Supplementary information

Self-supervised deep learning encodes high-resolution features of protein subcellular localization

In the format provided by the authors and unedited

Self-Supervised Deep Learning Encodes High-Resolution Features of Protein Subcellular Localization

Hirofumi Kobayashi[1], Keith C. Cheveralls[1], Manuel D. Leonetti[1], & Loic A. Royer[1]

Affiliations

[1]*Chan Zuckerberg Biohub, 499 Illinois Street, 94158 San Francisco, USA*

Supplementary Text

Interpreting the features as patterns in the images. An important and very active area of research in deep learning is the visualization, interpretation, and reverse-engineering of the inner working of deep neural networks[1–3]. To better understand the relationship between our input images and the emergent features obtained by *cytoself*, we conducted an experiment in which images were passed into the autoencoder while zeroing a given feature range before decoding. By computing the difference between the reconstructed images with or without zeroing, we identify specific regions of the images that are impacted, and thus causally linked, to that feature. Three examples are illustrated in Extended Data Fig. 9 and Supp. Fig. 7: (a) POLR2E, a core subunit shared between RNA polymerases I, II and III, (b) SEC22B, a vesicle-trafficking protein, and (c) RPS18, a ribosomal protein. For each protein we highlight (in red, Extended Data Fig. 9a-c) regions of the images that correspond to the three strongest peaks in their respective spectra. These difference maps reveal the image patterns that are lost and hence linked to that peak. The strongest peak (leftmost) of POLR2E's spectrum clearly corresponds to high intensity punctate structures within nucleoli, a localization recently established by Abraham *et al.*[4], while the two other peaks correspond to lower intensity and more diffuse patterns. In the case of SEC22B the strongest peak (leftmost) corresponds to cytoplasmic regions with high densities of vesicles. Other peaks in the spectrum of SEC22B correspond to regions with sparse punctate expression. Finally, for RPS18, the strongest peak (rightmost) corresponds to large, diffuse, and uniform cytoplasmic regions in the images, whereas the two other selected peaks correspond to brighter and more speckled regions (middle) as well as regions adjacent to the nuclear boundary (leftmost). This analysis highlights both the interpretability but also the high complexity of the encodings generated by our model.

Cropping based on fiducial channel centering versus content-based centering. Since the fiducial nuclear marker is used to centralize the input images around a nucleus it, is the marker necessary? To answer this question we trained *cytoself* on a dataset cropped on the basis of the image content alone (local image entropy) – forgoing the nuclear channel entirely. We compare the clustering scores obtained from this dataset with those obtained from the dataset cropped by centering nuclei and found the difference to be negligible (see Supp. Fig. 8). This result shows that the *texture* of the protein localization patterns is more important than the relative position of the fiducial marker to the protein fluorescence, or of its position within the cropped images. The main advantage of using the nuclear fiducial marker is to optimize the layout of the crops relative to the cells. Ideally we want to have one crop per cell, and one cell per crop. In contrast, random cropping without fiducial marker cannot ensure that every cell is used.

Dataset splitting into training, validation, and test sets. The training protocol described in the Methods section introduces data-leakage between training, validation and test data at pixel level. Another approach for splitting the data would be to split crops per field of view to ensure no that each pixel occurs only in one subset (train, validation or test). In the following we show that splitting our data along field of view does not change our results. We also explain why splitting the data into train-val-test sets is not as critical for self-supervised as it is for supervised learning.

First, we revisit our motivations for splitting the data in training, test, and validation sets. In a supervised setting, splitting the data in training, test, and validation sets serves two important purposes: (i) the test set is used to make an estimate of the performance of the model after supervised training, which is likely to generalize to further unseen data if it is *in-distribution*. (ii) the validation set is used during training to adjust the learning rate as well as to ensure early stopping to avoid over-fitting which could degrade performance on the test set. These considerations (i, ii, iii) are important for supervised learning. However, in our case, all training is self-supervised, and because the auto-encoder reconstruction and protein identification pretext *tasks* are not used *after* training and the performance metrics such as losses are not important for our end purpose. For the typical use-case of generating a feature vectors from input images, we never need to infer the identity of the tagged proteins nor do we need to reconstruct these images. While we do *not* use the pretext-tasks *per se* after training, we do use the resulting trained models and the latent representations that these models produce for given input images. Instead, we evaluated these models independently using our clustering score based on manually curated localization annotations. It follows then, that with our approach we could simply use the full dataset for training, without splitting the data. However, in general, it is often advantageous for technical purposes to do a train-val-test split to measure model convergence and detect over-fitting. The only disadvantage perhaps is that we could have trained *cytoself* on all of our data. In an abundance of caution, we use the test data for all analysis, but we could also have used the training data for the reasons explained above. Notwithstanding, it is in general preferable to avoid over-fitting, even in a self-supervised setting.

To ensure that our model does not overfit, we split our dataset *per field-of-view* and retrained the *cytoself* model. As shown in Supp. Fig. 9a, the gap between training and validation loss does not increase after about 120 epochs and 5 days of training, indicating that over-fitting does not occur. Another piece of evidence that our model did not over-fit to the training data is that our *cytoself* model actually works on images from the Allen Cell Collection (see Extended Data Fig. 8). One last point is to verify that indeed our results are not sensitive to the data-splitting method. First, we check whether the results of our ablation study still hold when splitting our dataset per field of view. To check this we recomputed the clustering scores. As shown in the Supp. Fig. 9b), the relative positions among these model variants stays roughly the same. Overall these results show that the different splitting scheme does not affect the relative performance between variants of our approach. Similarly, we recompute the UMAP in Fig. 2 of our manuscript and find no difference in how well clustered the data is (see Supp. Fig. 9c). Lastly, we redid the analysis on FAM241A and reach the same conclusion (see Supp. Fig. 9d). Overall, these results show that the technical choice of data splitting does not affect our results or conclusions.

Supplementary Figures

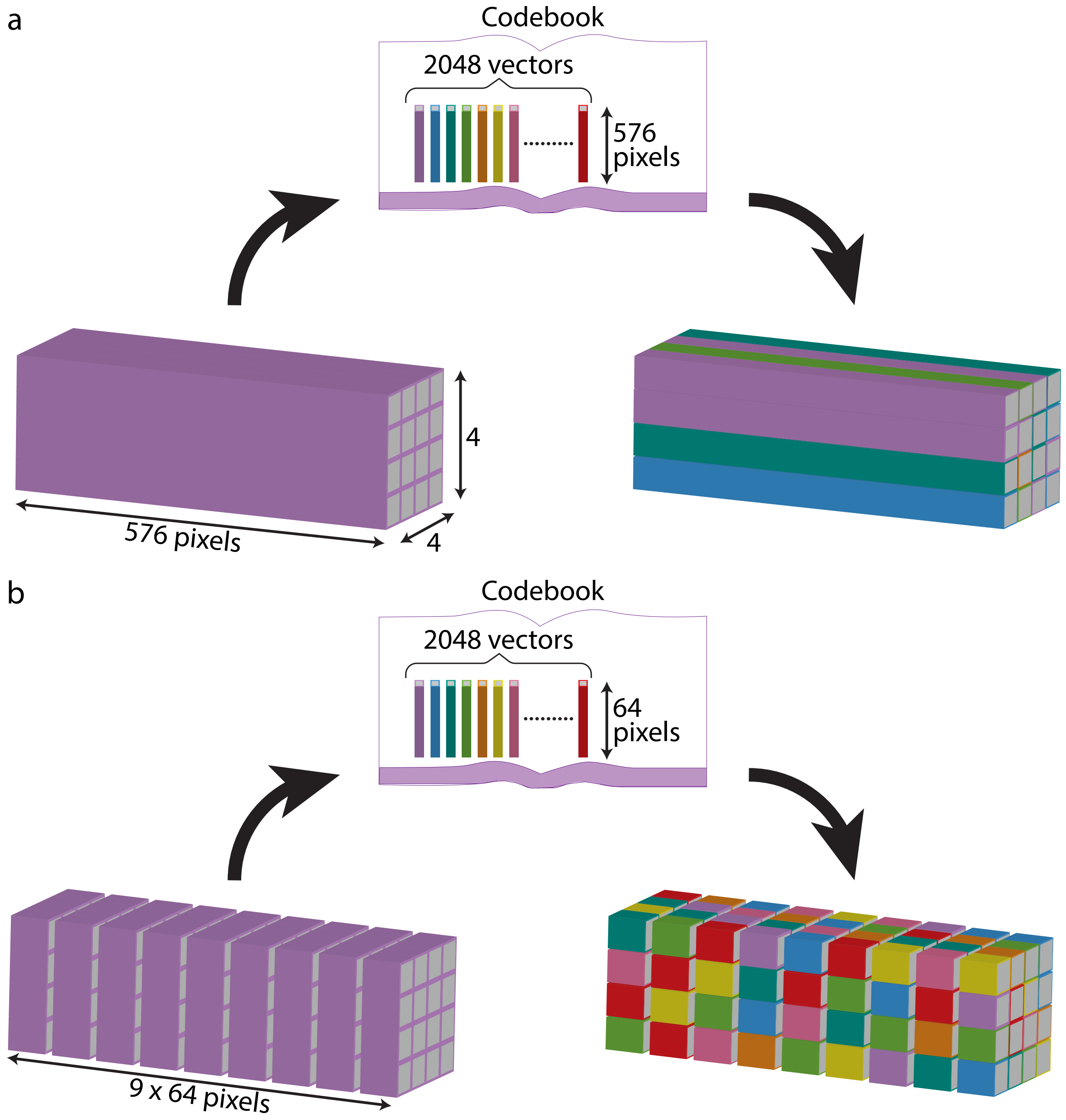

Supplementary Figure 1: A schematic of split quantization. **(a)**, Without split quantization, there are only $4 \times 4 = 16$ quantized vectors in the global representation. **(b)**, With split quantization, there are $4 \times 4 \times 9 = 144$ quantized vectors in the global representation, resulting in more opportunities for codes in the codebook to be used.

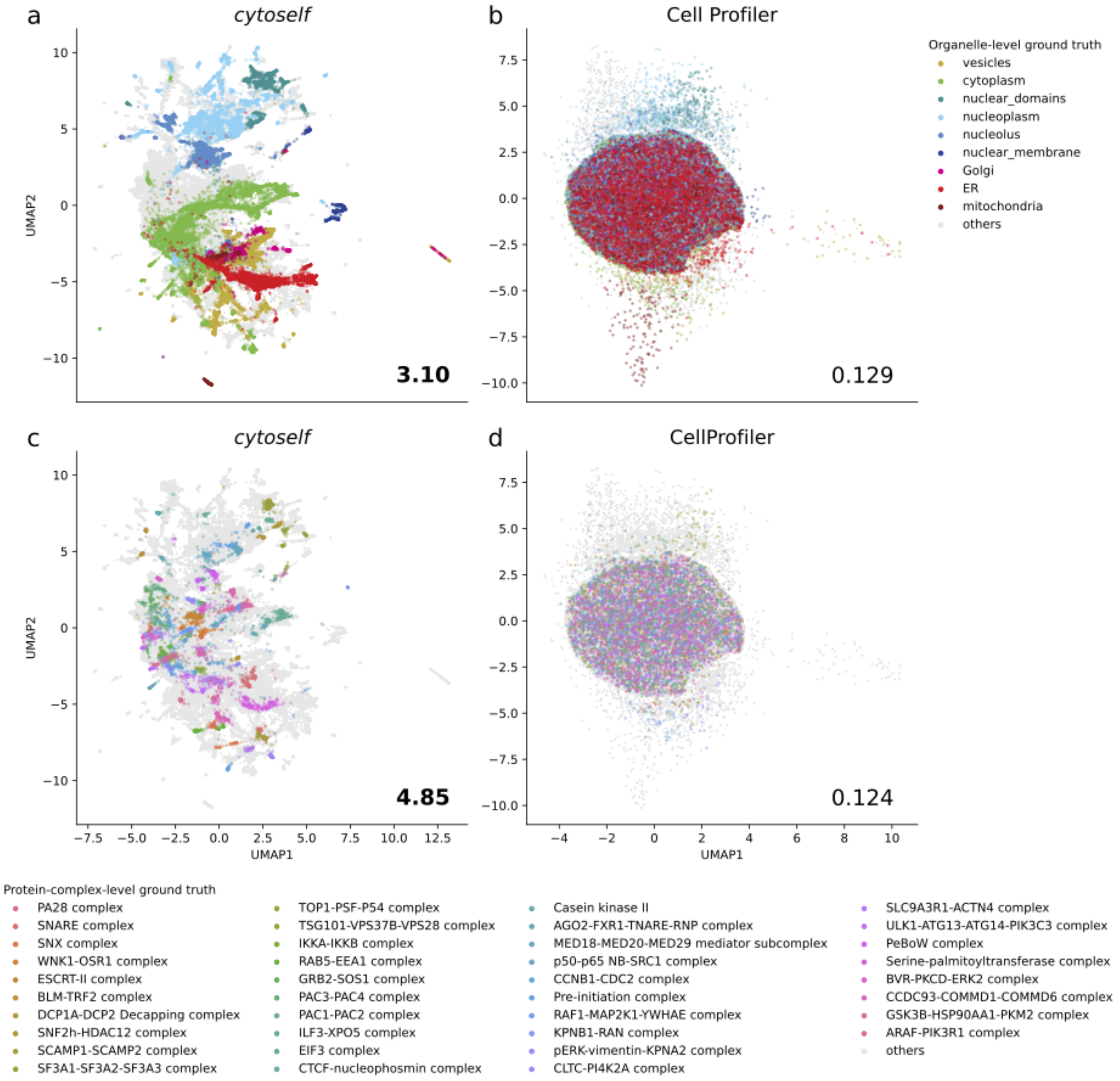

Supplementary Figure 2: Comparing *cytoself* with CellProfiler-derived image representations. Despite our best efforts (multiple attempts with different feature normalization schemes) CellProfiler features lead to very poor clustering score (< 0.13) versus *cytoself* (> 3). **(a)** UMAP using cytoself features annotated with organelle-level ground truth. **(b)** UMAP using Cell Profiler features annotated with organelle-level ground truth. **(c)** UMAP using cytoself features annotated with protein-complex-level ground truth. **(d)** UMAP using Cell Profiler features annotated with protein-complex-level ground truth.

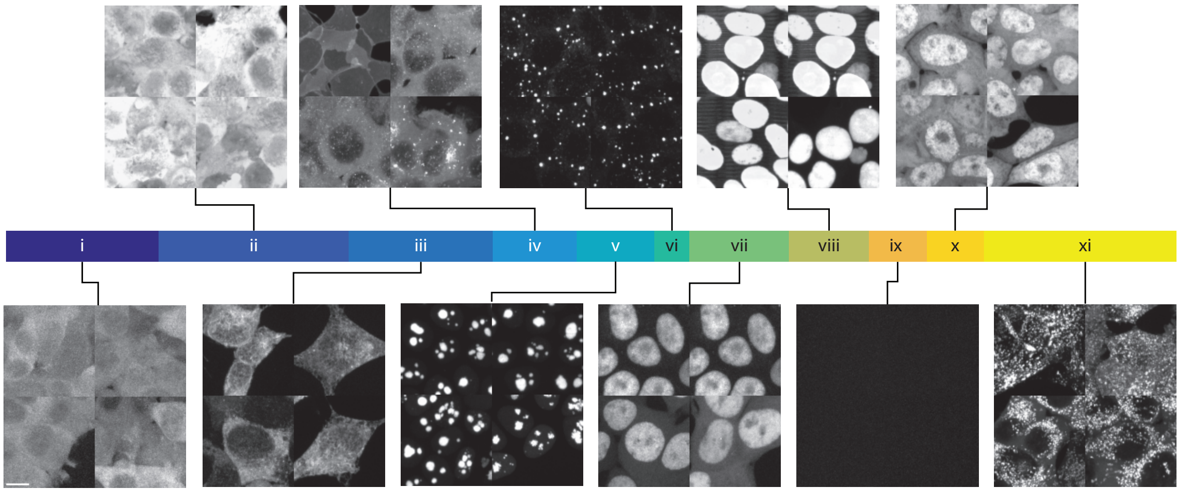

Supplementary Figure 3: Representative images for the 11 top-level clusters. We show representative images for all 11 clusters and the corresponding localizations categories (i) cytoplasmic/membrane, (ii) cytoplasmmic/nucleoplasm, (iii) ER, (iv) membrane, (v) nucleolus, (vi) vesicles, (vii) nucleoplasm, (viii) nucleoplasm, (ix) unsuccessful image, (x) cytoplasmic/nucleoplasm, (xi) vesicles. The images in each category have the highest occurrence of the corresponding features (see Fig. 5a).

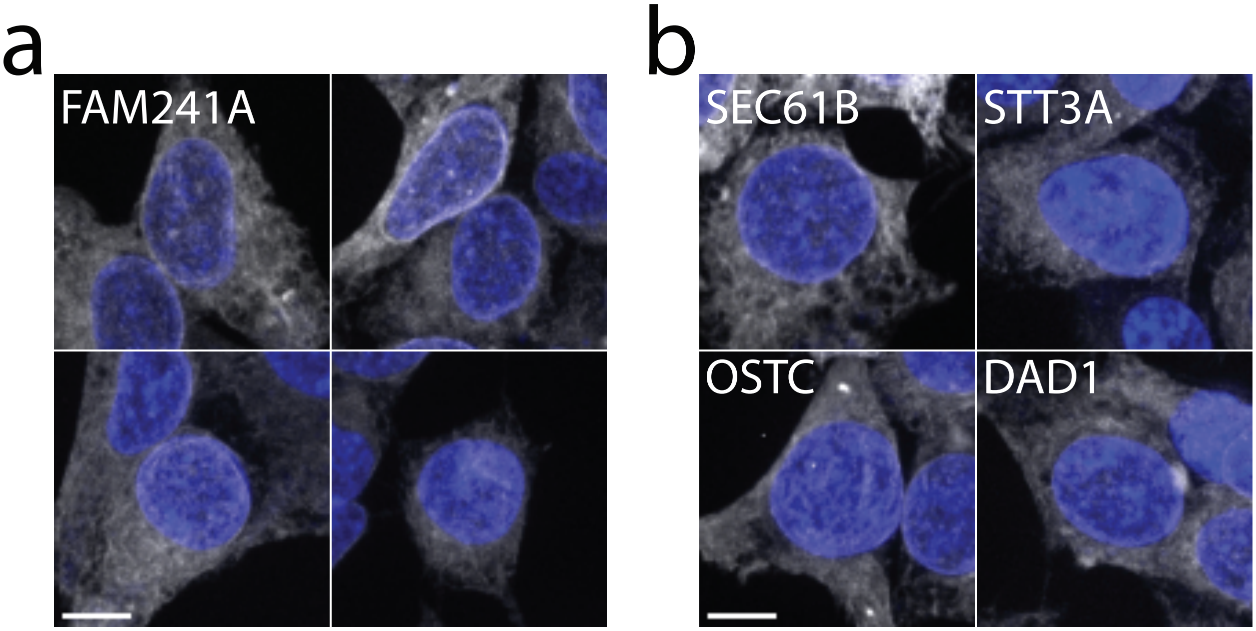

Supplementary Figure 4: Fluorescence images for FAM241A **(a)** versus representative images of other ER localized proteins **(b)**. Protein localization and nuclei are displayed in gray and blue respectively. Images were randomly selected from each protein. Scale bars: $10\mu m$

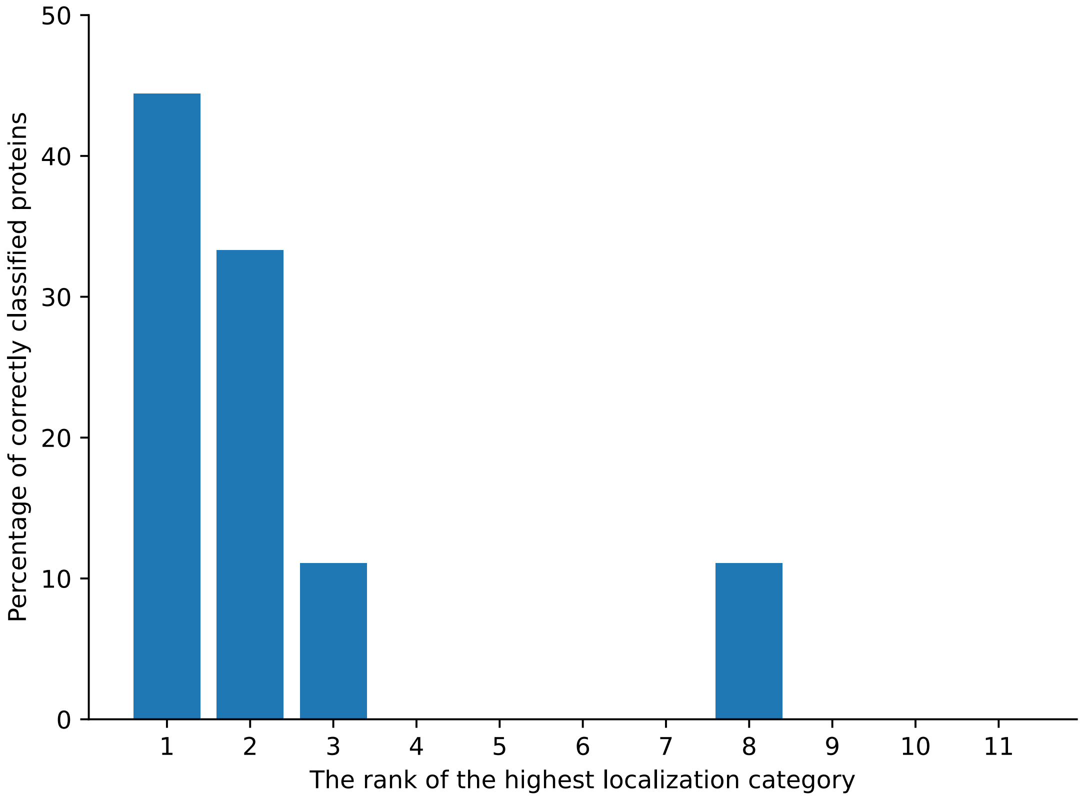

Supplementary Figure 5: Comparing the predicted localization categories of proteins present both in OpenCell and the Allen Institute dataset. We find 9 proteins in the intersection between the two datasets (9 out of 11 in the Allen dataset). We compute the feature spectra from images from the Allen dataset, predict the corresponding localization categories, and compare these to the predictions done on the basis of the OpenCell images. Localization categories are predicted in the same way as done for FAM241A.

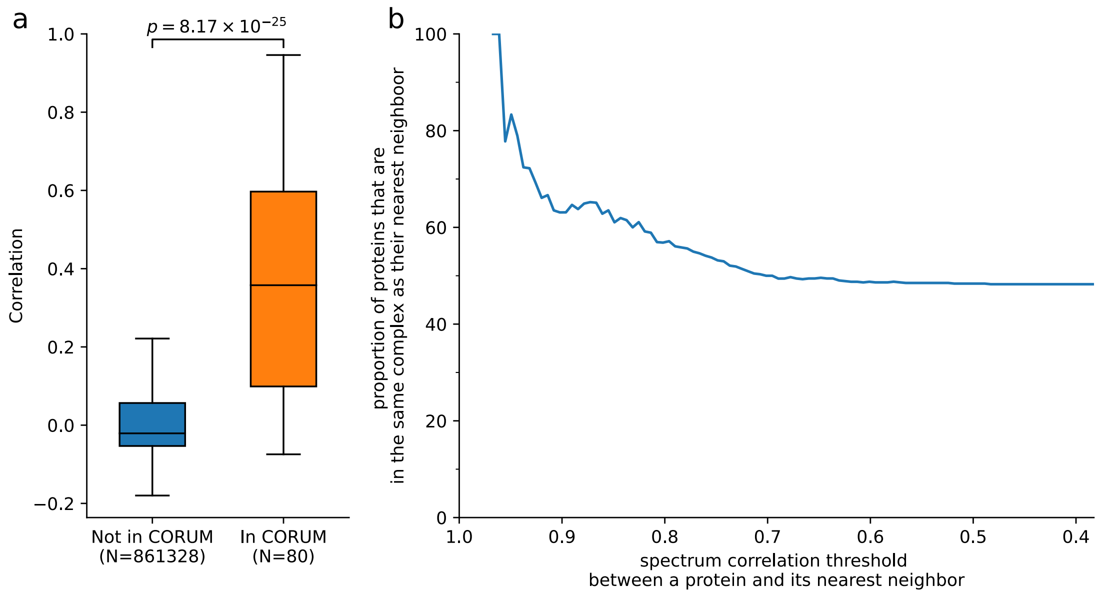

Supplementary Figure 6: Highly correlated spectra imply shared protein complex membership. **(a)** Feature spectra of protein pairs that are in the same complex according to CORUM[5] show significantly higher correlations than those that are not in the same complex, confirming quantitatively that the feature spectra are sensitive enough to encode complex-specific patterns. However, the spread in correlations also indicates that not all interacting proteins have strongly correlated spectra which is expected when considering that proteins can participate in different protein complexes and thus exhibit mixed localizations. In contrast, the correlation of feature spectra for protein pairs that are not in CORUM are typically close to zero with less spread, suggesting that it is rare for non-interacting proteins to have highly correlated spectra. **(b)** We plot the proportion of proteins in both OpenCell and CORUM that share protein-complex membership with their most correlated neighbor. When we consider only correlations above a threshold of 0.95 we find that in 83.3% of cases the protein with the strongest correlation is in a shared complex. For a threshold of 0.90 the value is 66.3%, and for a threshold of 0.5 the value is 47.9%. Each box indicates the extent from the first to the third quartile of the data, with a line representing the median. The whiskers indicates 1.5 times the inter-quartile range. The p-value is computed using a two-sided Mann-Whitney U test.

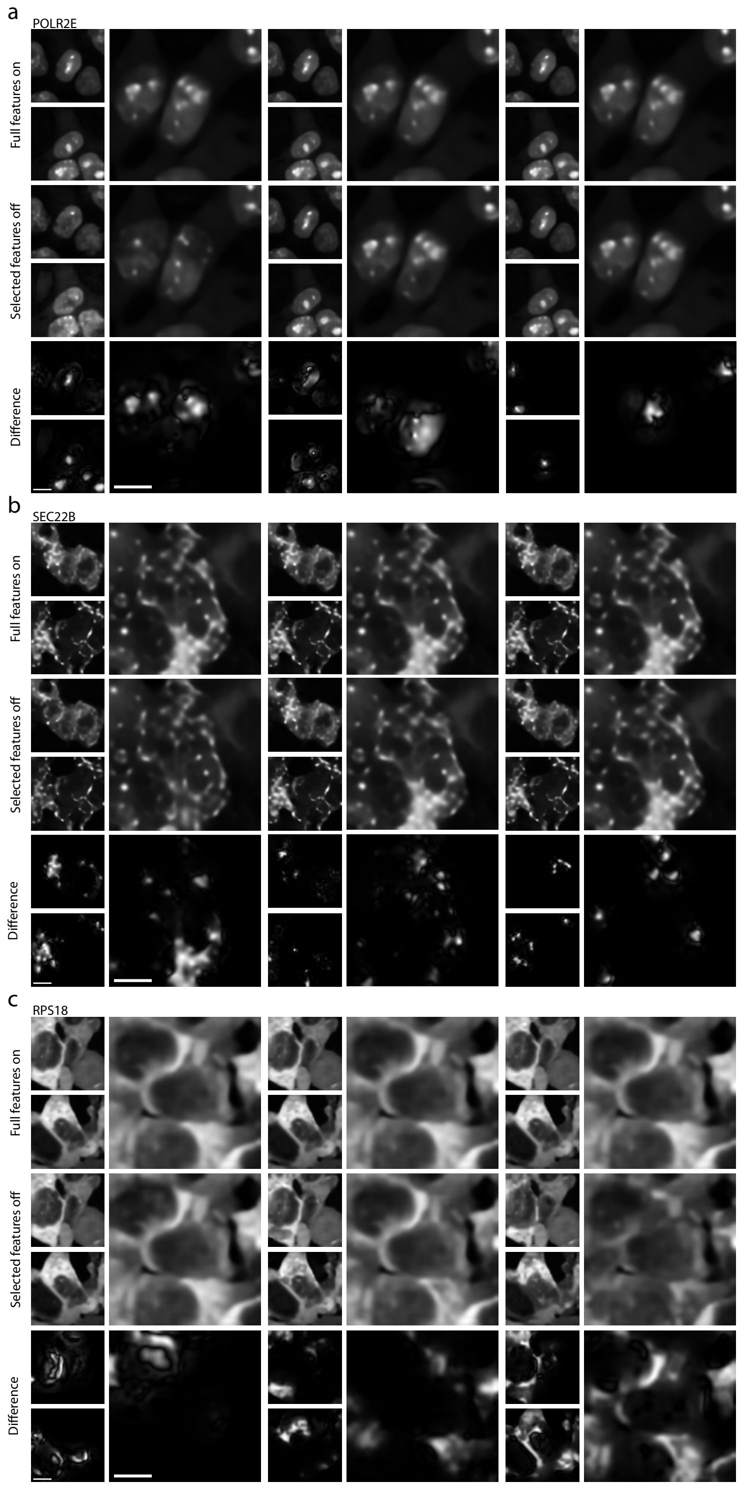

Supplementary Figure 7: Reconstructed images with full features on, specific features off and the differences. Each panel and column correspond to those in Extended Data Fig. 9. The pixel intensities are rescaled to the minimum and maximum of each image. Scale bars: $10\mu m$

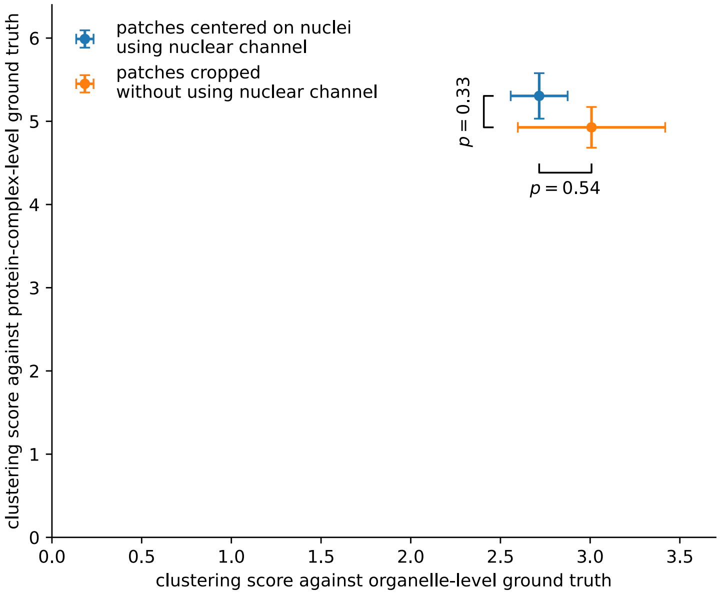

Supplementary Figure 8: Clustering performance of *cytoself* is not significantly affected by using a training dataset cropped with or without using the nuclear fiducial channel. We avoid the use of the fiducial marker by: extracting a large number of random crops from the images, computing the histogram of each crop, computing the entropy of each of these histograms, sorting the crops by entropy, and keeping the top half of highest entropy. Variance statistics were obtained by training model variants 5 times followed by computing UMAP 10 times per trained model. The p-values are computed using two-sided Student's t-test.

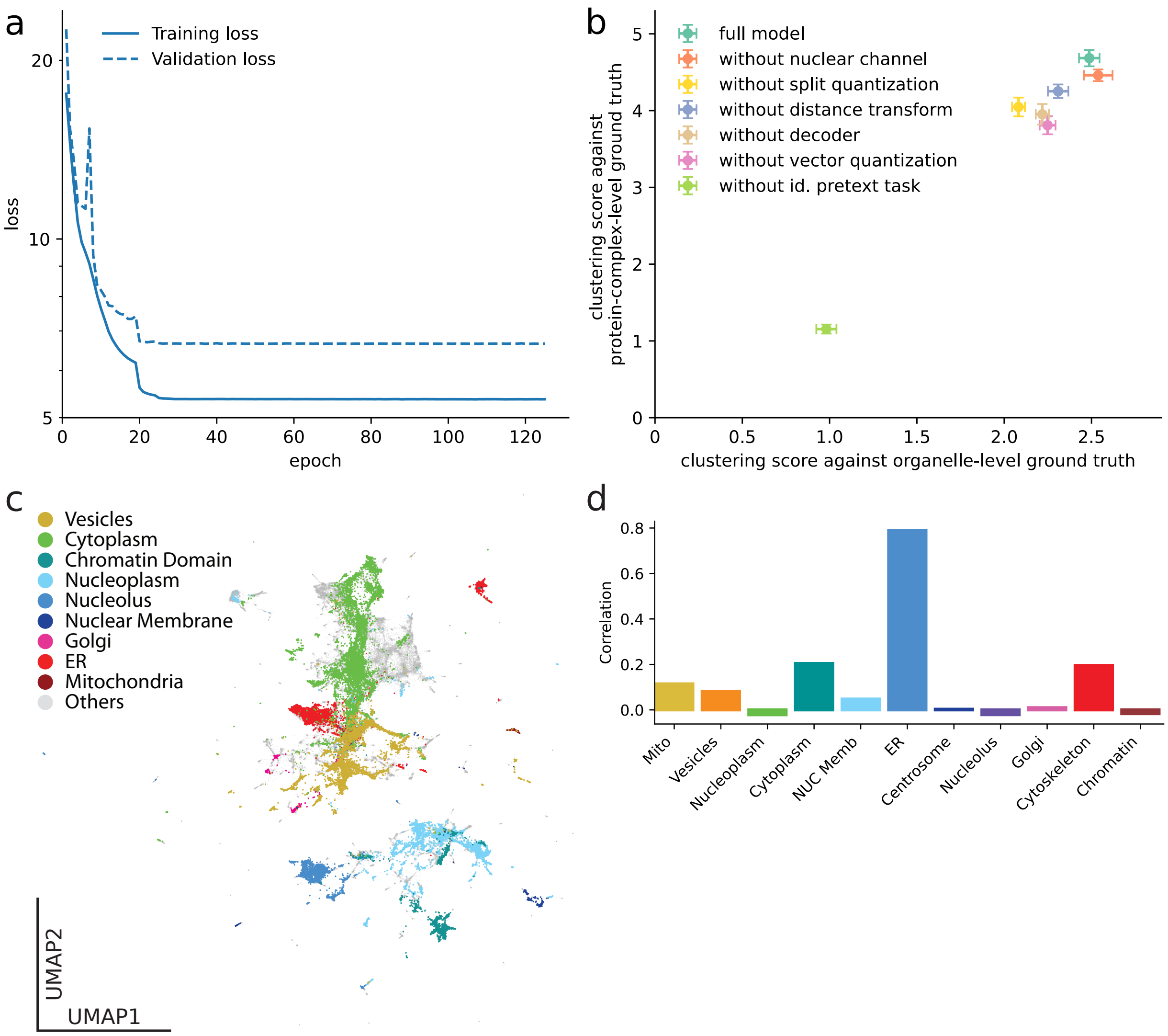

Supplementary Figure 9: Training *cytoself* on image crops split by fields of view does not affect our results and conclusions. Splitting by fields of view ensures that each pixel occurs only once in the training data, validation data or test data, exclusively. **(a)** Training history on datasets split on fields of view showing that no over-fitting occurs even after more than 120 epochs. **(b)** Clustering scores obtained from datasets split on fields of view show that the conclusions of our ablation study are unchanged. **(c)** The appearance of the UMAP shown in Fig. 2 is not fundamentally affected by splitting on fields of view. **(d)** Splitting by fields of view does not affect our result that FAM241A is localized in the ER.

Supplementary Files

1. model_structures.zip, Detailed structure of VQ-VAE model, including **(a)** the whole model structure, **(b)** the structure of encoder1, **(c)** the structure of encoder2, **(d)** the structure of decoder1, **(e)** the structure of decoder2.
2. proteins_uniorg.csv, The ground truth used for evaluating clustering performance at organelle level.
3. proteins_corum.csv, A list of protein subunits collected from CORUM[5] as a ground truth to compute clustering scores. See Methods for how they were selected.
4. proteins_uniloc.csv, The ground truth used for evaluating feature spectra.
5. proteins_subunits.csv, A list of protein subunits for protein complexes mentioned in Fig. 2 and Fig. 3b.

References

1. Yosinski, J., Clune, J., Nguyen, A., Fuchs, T. & Lipson, H. Understanding neural networks through deep visualization. *arXiv preprint arXiv:1506.06579* (2015).

2. Montavon, G., Samek, W. & Müller, K.-R. Methods for interpreting and understanding deep neural networks. *Digital Signal Processing* **73**, 1–15 (2018).

3. Zaritsky, A. *et al.* Interpretable deep learning uncovers cellular properties in label-free live cell images that are predictive of highly metastatic melanoma. *Cell Systems* **12**, 733–747 (2021).

4. Abraham, K. J. *et al.* Nucleolar rna polymerase ii drives ribosome biogenesis. *Nature* **585**, 298–302 (2020).

5. Giurgiu, M. *et al.* Corum: the comprehensive resource of mammalian protein complexes—2019. *Nucleic acids research* **47**, D559–D563 (2019).

6. Wu, H. & Flierl, M. Vector quantization-based regularization for autoencoders. *Proceedings of the AAAI Conference on Artificial Intelligence* **34**, 6380–6387 (2020).

7. Lu, A. X., Kraus, O. Z., Cooper, S. & Moses, A. M. Learning unsupervised feature representations for single cell microscopy images with paired cell inpainting. *PLoS computational biology* **15**, e1007348 (2019).

8. Schröder, B. A., Wrocklage, C., Hasilik, A. & Saftig, P. The proteome of lysosomes. *Proteomics* **10**, 4053–4076 (2010).

9. Gosney, J. A., Wilkey, D. W., Merchant, M. L. & Ceresa, B. P. Proteomics reveals novel protein associations with early endosomes in an epidermal growth factor–dependent manner. *Journal of Biological Chemistry* **293**, 5895–5908 (2018).
