# Supplementary material for: Self-supervised deep learning encodes high-resolution features of protein subcellular localization
Source: Nat Methods. 2022 Jul 25;19(8):995–1003. doi: 10.1038/s41592-022-01541-z (PMC9349041; doi:10.1038/s41592-022-01541-z)
Supplement: Supplementary file 2 — Reporting Summary. [file 41592_2022_1541_MOESM2_ESM.pdf]

Corresponding author(s): Loic A. Royer, Hirofumi Kobayashi, Manuel Leonetti

Last updated by author(s): Apr 27, 2022

Reporting Summary

Nature Research wishes to improve the reproducibility of the work that we publish. This form provides structure for consistency and transparency in reporting. For further information on Nature Research policies, see our Editorial Policies and the Editorial Policy Checklist.

Statistics

For all statistical analyses, confirm that the following items are present in the figure legend, table legend, main text, or Methods section.

n/a	Confirmed	
☐	☒	The exact sample size (*n*) for each experimental group/condition, given as a discrete number and unit of measurement
☐	☒	A statement on whether measurements were taken from distinct samples or whether the same sample was measured repeatedly
☐	☒	The statistical test(s) used AND whether they are one- or two-sided<br>*Only common tests should be described solely by name; describe more complex techniques in the Methods section.*
☒	☐	A description of all covariates tested
☐	☒	A description of any assumptions or corrections, such as tests of normality and adjustment for multiple comparisons
☐	☒	A full description of the statistical parameters including central tendency (e.g. means) or other basic estimates (e.g. regression coefficient) AND variation (e.g. standard deviation) or associated estimates of uncertainty (e.g. confidence intervals)
☐	☒	For null hypothesis testing, the test statistic (e.g. *F*, *t*, *r*) with confidence intervals, effect sizes, degrees of freedom and *P* value noted<br>*Give P values as exact values whenever suitable.*
☒	☐	For Bayesian analysis, information on the choice of priors and Markov chain Monte Carlo settings
☒	☐	For hierarchical and complex designs, identification of the appropriate level for tests and full reporting of outcomes
☒	☐	Estimates of effect sizes (e.g. Cohen's *d*, Pearson's *r*), indicating how they were calculated

Our web collection on statistics for biologists contains articles on many of the points above.

Software and code

Policy information about availability of computer code

Data collection	Data per se was not collected in this study.
Data analysis	The source code is available at https://github.com/royerlab/cytoself

For manuscripts utilizing custom algorithms or software that are central to the research but not yet described in published literature, software must be made available to editors and reviewers. We strongly encourage code deposition in a community repository (e.g. GitHub). See the Nature Research guidelines for submitting code & software for further information.

Data

Policy information about availability of data

All manuscripts must include a data availability statement. This statement should provide the following information, where applicable:

- Accession codes, unique identifiers, or web links for publicly available datasets
- A list of figures that have associated raw data
- A description of any restrictions on data availability

The image data used in this work is available at https://github.com/royerlab/cytoself.
The CORUM database is independently available at: http://mips.helmholtz-muenchen.de/corum/
Image data from the Allen Institute is independently available at: https://www.allencell.org/data-downloading.html#DownloadImageData

Field-specific reporting

Please select the one below that is the best fit for your research. If you are not sure, read the appropriate sections before making your selection.

☒ Life sciences ☐ Behavioural & social sciences ☐ Ecological, evolutionary & environmental sciences

For a reference copy of the document with all sections, see nature.com/documents/nr-reporting-summary-flat.pdf

Life sciences study design

All studies must disclose on these points even when the disclosure is negative.

Sample size	We used all available data from OpenCell database, in which 1311 proteins were tagged and 24,382 fluorescence images were acquired.
Data exclusions	No data was excluded.
Replication	All the deep learning models were trained for 5 times. All the UMAPs were computed for 10 times.
Randomization	The randomization happens internally when training a deep learning model or computing UMAP. For other analysis, we used all available data and did not need randomization.
Blinding	We trained the cytoself model by holding out one protein.

Reporting for specific materials, systems and methods

We require information from authors about some types of materials, experimental systems and methods used in many studies. Here, indicate whether each material, system or method listed is relevant to your study. If you are not sure if a list item applies to your research, read the appropriate section before selecting a response.

Materials & experimental systems

n/a	Involved in the study	
☒	☐	Antibodies
☐	☒	Eukaryotic cell lines
☒	☐	Palaeontology and archaeology
☒	☐	Animals and other organisms
☒	☐	Human research participants
☒	☐	Clinical data
☒	☐	Dual use research of concern

Methods

n/a	Involved in the study	
☒	☐	ChIP-seq
☒	☐	Flow cytometry
☒	☐	MRI-based neuroimaging

Eukaryotic cell lines

Policy information about cell lines

Cell line source(s)	ATCC CRL-3216
Authentication	Short Tandem Repeat Analysis (cell culture facility, UC Berkeley)
Mycoplasma contamination	Mycoplasma contamination: Cell lines were tested negative for mycoplasma (MycoAlert Plus, Lonza)
Commonly misidentified lines (See ICLAC register)	None
