# Supplementary figures and images for: Self-supervised deep learning encodes high-resolution features of protein subcellular localization
Source: Nat Methods. 2022 Jul 25;19(8):995–1003. doi: 10.1038/s41592-022-01541-z (PMC9349041; doi:10.1038/s41592-022-01541-z)

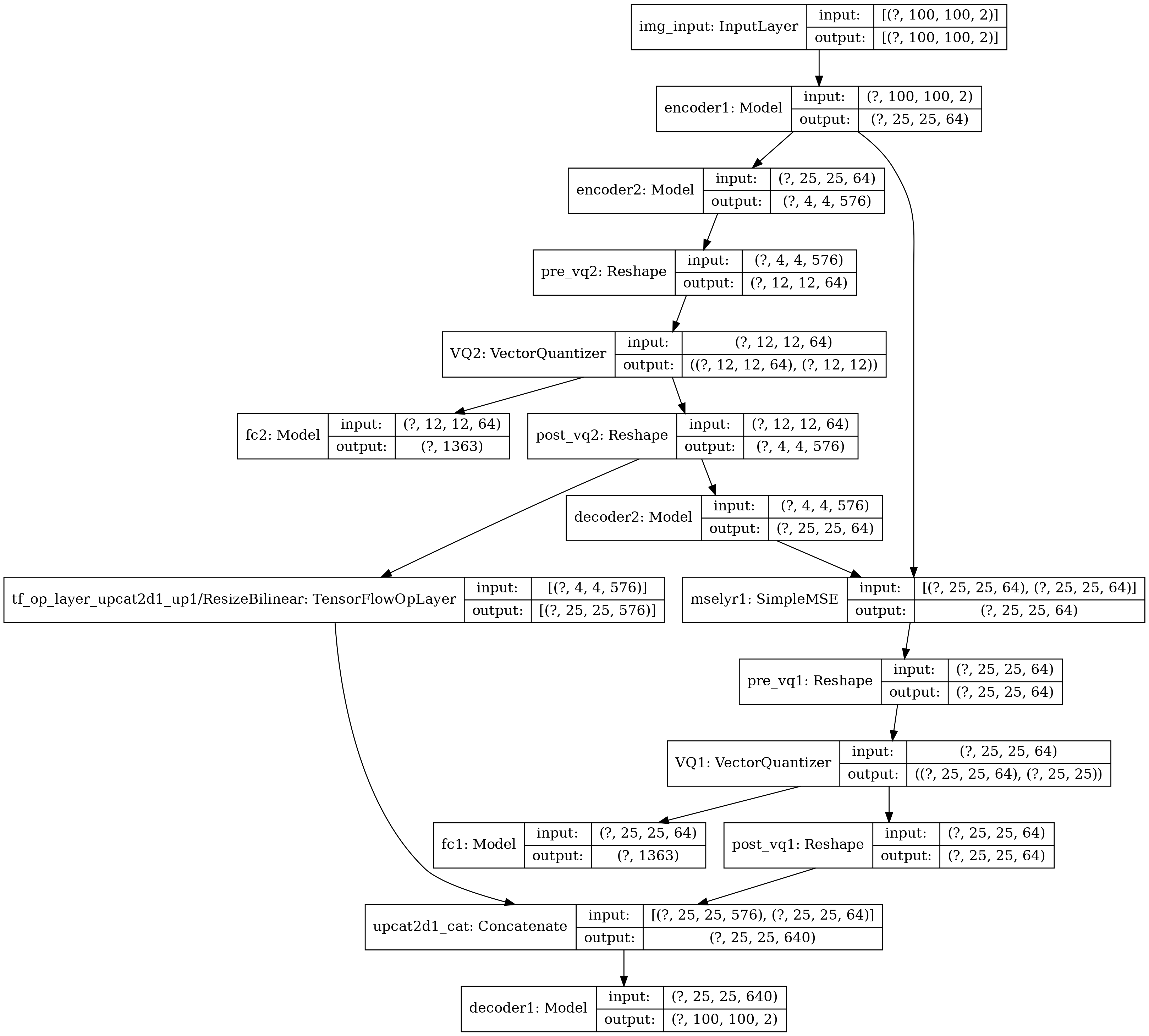

Supplement: Supplementary file 3 — a–e, Detailed structure of VQ-VAE model, including the whole model structure (a), the structure of encoder1 (b), the structure of encoder2 (c), the structure of decoder1 (d) and the structure of decoder2 (e). [file 41592_2022_1541_MOESM3_ESM.zip › a_whole_model.png]

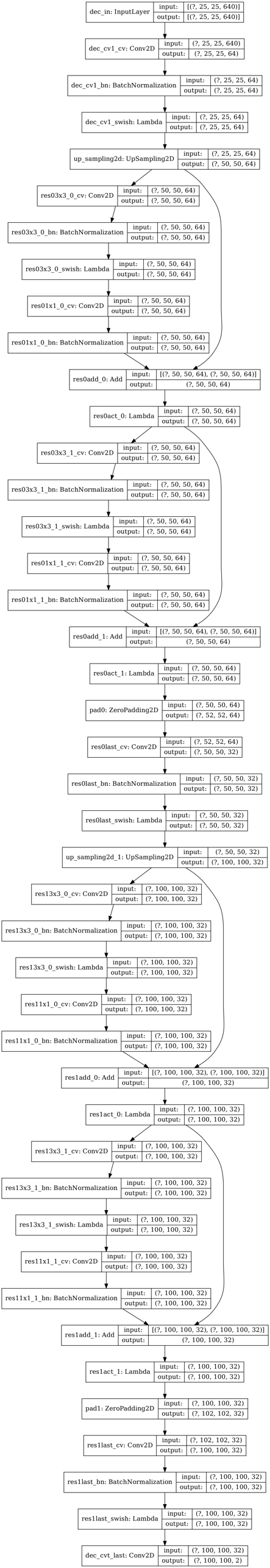

Supplement: Supplementary file 3 — a–e, Detailed structure of VQ-VAE model, including the whole model structure (a), the structure of encoder1 (b), the structure of encoder2 (c), the structure of decoder1 (d) and the structure of decoder2 (e). [file 41592_2022_1541_MOESM3_ESM.zip › d_decoder1.png]
